# Assessment of indoor and outdoor air quality in primary schools of Cyprus during the COVID–19 pandemic measures in May–July 2021

**DOI:** 10.1016/j.heliyon.2022.e09354

**Published:** 2022-05-02

**Authors:** Corina Konstantinou, Andria Constantinou, Eleni G. Kleovoulou, Alexis Kyriacou, Christina Kakoulli, George Milis, Michalis Michaelides, Konstantinos C. Makris

**Affiliations:** a Cyprus International Institute for Environmental and Public Health (CII), Cyprus University of Technology, Limassol, Cyprus; b LELANTUS Innovations Ltd, Nicosia, Cyprus; c PHOEBE Research & Innovation Ltd, Nicosia, Cyprus; d Department of Electrical Engineering, Computer Engineering and Informatics, Cyprus University of Technology, Limassol, Cyprus

**Keywords:** Indoor air quality, School, Environmental pollutants, Sensors, Exposome, COVID–19 measures

## Abstract

Combined pollutant effects from indoor and outdoor sources on children's health, while being at school have not been holistically tackled. The aim of the School Temperature and Environmental Pollutants Study (STEPS) was to perform a school population representative assessment of indoor air quality (IAQ) in primary schools of densely and intermediate populated areas of Cyprus (n = 42). The study took place during May–July 2021 when a school-specific COVID-19 protocol was in place. Questionnaire-based characteristics of schools/classrooms were collected along with 24/48-h long IAQ monitoring of air temperature, relative humidity (RH), particulate matter (PM), carbon dioxide (CO_2_) and volatile organic compounds (VOCs), using low-cost sensors. Mixed effect models assessed the IAQ determinants during school hours. Indoor PM, temperature, RH and VOCs increased with progressing school periods in the day, while indoor CO_2_ decreased. Indoor RH and CO_2_ were negatively associated with % open windows, while indoor PM_2.5_ was positively associated. Most of school time (85%), indoor air temperature exceeded the recommended upper limit (27 °C), while a third of indoor PM_2.5_ (24-h) measurements exceeded 15 μg/m^3^. The interplay of clean indoor air with adequate ventilation and adaptation to heat stress in schools is important and its comprehensive characterization requires holistic methodological approaches and tools.

## Highlights

• A primary school population representative study of indoor air quality was conducted in Cyprus during May-July 2021.
• Natural ventilation measures, like open windows and doors during class hours, helped in maintaining adequate ventilation.
• The study took place during the summer period with indoor air temperature being above the recommended value most of school time.
• A third of the 24-hour indoor PM_2.5_ measurements exceeded the WHO recommended value.

**Practical implications**

Strong scientific evidence exists for outdoor air pollution health adverse effects; however, little is known on the combined effects of sources and determinants of indoor air quality (IAQ) on human health, and this holds true also for the school environment. Our school population representative study in Cyprus focused on the IAQ dynamics of primary schools using the methodological framework of the human exposome during the heat-stressed period of May–July, while a COVID-19 school protocol was in place. Natural ventilation measures of systematically keeping open windows and doors during class hours helped in maintaining adequate ventilation, however, the vast majority of classrooms had air temperatures above recommended values and a third of indoor PM_2.5_ values exceeded the 24h-recommended value. Further research is warranted to better understand the interplay and dynamics of inter-correlated IAQ parameters in association with climate and health outcomes or indicators of disease spread and control, including policy actions that respond to climate crisis.

## 1 Introduction

Children in schools are exposed to a suite of environmental pollutants from both indoor and outdoor sources [1, 2]. Children's unique characteristics in terms of their metabolism, behaviour, physiology, growth and development make them especially vulnerable to poor environmental conditions [3, 4]. These conditions might be a significant determinant of health in their later life [5].

Children spend around 25–30% of their time at school during weekdays [6, 7], and indoor environmental conditions are among the major contributors of their total body burden to various air pollutants [3, 8, 9]. During the last two decades, particulate matter (PM), one of the main air pollutants, was found to be in high concentrations in many schools across different regions of the world [2]. The recent SINPHONIE (Schools indoor pollution and health: Observatory network in Europe) project showed that schoolchildren in 23 European countries were exposed to PM_2.5_, radon and carbon dioxide (CO_2_) [11], in higher concentrations than the thresholds defined by the air quality guidelines of the World Health Organization (WHO) [12]. The same study found that the average daily indoor air temperature across schools was 22 °C (SD: 2.11) [11], lying within the recommended range of 15–25 °C, defined by the WHO for indoor air temperatures for minimum energy expenditure [13]. Air temperature in school classrooms in Singapore was as high as 29.5 °C [14], whereas the indoor air temperature of a classroom in China was as low as 14.7 °C [15].

Indoor air temperature is an integral component of “thermal comfort”, which is defined by the American Society of Heating, Refrigerating and Air–Conditioning Engineers (ASHRAE) as the “condition of mind which expresses human satisfaction with the thermal environment” [16]. Indoor overheating experienced at mean temperatures above 25 °C [13], is an important phenomenon that warrants attention especially in school classrooms, along with other environmental stressors, including poor ventilation; these are well known stressors directly or indirectly associated with student performance, attention and children's health [1, 12]. Typically, the indoor environment is studied in combination with the outdoor air characteristics, since the indoor conditions are highly influenced by the outdoor conditions. More specifically, the continuous interaction of indoor and outdoor spaces through windows, doors and other openings, create strong interconnections in the dynamics of all air quality parameters. Hence, assessment of both indoor and outdoor air quality parameters in schools is equally important for more accurate conclusions [10]. Therefore, the comprehensive assessment of children's exposomes, i.e., the sum of their environmental exposures during childhood would entail a comprehensive assessment of key indoor and outdoor environmental stressors impacting on schools' indoor air quality (IAQ). The systematic collection of measurements for a suite of environmental parameters in school classrooms can be performed using appropriate sensing and monitoring infrastructure and the methodological framework of the human exposome [17]. The human exposome framework is used in environmental health sciences and allows for simultaneously assessing a suite of environmental stressors in schools, including the consideration of relevant health and wellbeing or climate adaptation policies and programs [18].

Cyprus is a Mediterranean island, considered as a hot spot for manifestations of climate change. Daily outdoor air temperature measurements in selected stationary points around the country are available; for example, daily outdoor air temperatures in the city of Limassol from May to September 2020 varied between 12 °C and 41 °C [19]. Similar measurements of the indoor air temperature variation and magnitude within schools in Cyprus are not available. Thus, it is difficult to assess children's exposures during school class hours, i.e., about ∼6 h every weekday. The IAQ attracted more attention during the COVID–19 pandemic period because schools were considered as settings of possible virus transmission [20]. Given the high air temperatures observed during May–September in the last years in Cyprus [19] and the fact that no specific guidelines are in place for IAQ at schools, the aim of the School Temperature and Environmental Pollutants Study (STEPS) was to perform a comprehensive assessment of IAQ in primary schools in Cyprus during the COVID-19 pandemic period in May–July 2021, when a school-specific COVID-19 protocol was in place.

Thus, the objectives of the STEPS study were: i) to describe the magnitude and variance of a suite of environmental parameters [air temperature, Relative Humidity (RH), Particulate Matter (PM), and chemicals [Carbon Dioxide (CO_2_); Volatile Organic Compounds (VOCs)] in classrooms of public primary schools located in densely and intermediately populated areas of Cyprus; ii) to compare the magnitude of the environmental stressors indoors versus outdoors; and iii) to compare the variability of the IAQ metrics against recommended cutoff values by international authoritative organizations.

## 2 Methods

### 2.1 Study design and COVID–19 health protocol in schools

A school population representative, cross sectional study was set up in public primary schools of densely and intermediate populated areas (degree of urbanization 1 and 2), in the five districts of the government–controlled areas of the Republic of Cyprus (Limassol, Nicosia, Larnaca, Pafos, Famagusta). The study protocol was approved by the Cyprus National Bioethics Committee (2021.01.98) and the Cyprus Ministry of Education, Culture, Youth and Sports (MECYS) (7.15.06.12/4).

The study took place during a continuous 9–week period (May 10 – July 7, 2021) when a strict COVID–19 pandemic protocol designed for schools was in place by the Republic of Cyprus [21]. This COVID–19 protocol for the primary schools of Cyprus included, among others, measures regarding mask use, distance, occupancy and cleaning/disinfection. Specifically, it recommended the use of masks among children in classrooms and the washing of hands with antiseptic when entering and exiting the classroom. Desks should be spaced 1 m apart, with students sitting in prespecified seats, windows and doors should be open, while the number of students in each class should not exceed 12. Recommendations also included daily cleaning and disinfection of classrooms with chlorine, while constant natural aeration during and between teaching periods should take place, to the extent possible.

### 2.2 Selection of schools and sample size

Prior to randomization, a list of eligible schools was created combining the list of all currently operating schools in Cyprus and the most updated list with the degree of urbanization per municipality/village of Cyprus. From this list, 42 schools in municipalities/villages with degree of urbanization 1 or 2 (densely and intermediate populated areas) were randomly selected to participate in the study. According to the available information from the Ministry of Education, Culture, Youth and Sports [22], 126 of 330 public schools were located in the major Cypriot cities and were potentially eligible to participate in the study. The number of schools in areas with degree of urbanization 1 or 2 was similar and, thus, the selection of 42 schools ensured that 13% of all public schools and 33% of the eligible schools would be selected for this study.

Moreover, it was estimated that a sample size of 36 schools would allow to detect differences in the air temperature of minimum 1 °C in the two settings (indoors and outdoors) with a standard deviation of 2.1 °C [11], with 80% power at 5% significance level. Therefore, a random sample of 42 schools would allow the study to be conducted within a strict timeline, being representative of schools in urban areas, and having the power to detect indoor vs. outdoor differences in mean levels of environmental parameters.

### 2.3 Data collection

The methodological approach followed was based on the SINPHONIE project, which among other objectives, focused on improving IAQ assessment in European schools [23].

The first step in the project was to obtain the necessary approvals for the study. Immediately after securing these approvals by the authorities, we contacted the respective school boards and then the school headmasters of the selected schools. The aim of this first contact was to explain the objectives of the study and the process of the equipment installation and removal, as well as to ask for permission to enter the school premises and arrange appointments for the equipment installations.

A suite of exposomic tools, ranging from questionnaires together with low-cost sensors for monitoring different physical, particulate and chemical parameters, were deployed in school classrooms operating under a COVID-19 health protocol. Regarding the collection of IAQ measurements, we placed sensors at three locations per school, one outdoor and two indoor in classrooms. Two types of sensors were installed in each location for 24 or 48 h at approximately 1.50–2 m height from the floor and away from the writing board (to avoid effects of the chemicals of writing instruments). Two classrooms in each school were selected as “sub–school” sampling locations to account for within–school differences in the measured environmental parameters.

The classrooms were selected based on the team's assessment in the field and after discussion with the headmaster and the teachers to ensure that the sampling would both be adequate to fulfill the study objectives while at the same time not being disruptive to the school activities. The teachers/headmasters were asked to report whether they had noticed any issues in a specific classroom with regards to IAQ e.g., recent complaints about specific annoyances, such as higher air temperature or a recent event linked to poor IAQ, such as a known transmission in the classroom of flu or SARS–CoV-2. Classrooms for which teachers reported such concerns (atypical) were selected for sampling and comparison to ensure that the classroom selection accounted for site–specific characteristics.

The outdoor sensors were placed at a shaded area and if a nearby power source was not available, dedicated power-bank devices were used to secure powering of the sensors for the measurements’ collection duration.

A study log was maintained per school, during the installation visit and included information regarding the sensors’ IDs used in each school location, characteristics of the classrooms (i.e., windows type, number and direction, classroom area), as well as other general observations that might impact the interpretation of the results of the study.

### 2.4 Sensors

PurpleAir sensors (PurpleAir, Draper, Utah, USA) were used to measure PM_10_, PM_2.5_ and PM_1_ and collected 2-minute signals in an SD card [25]. MCF88 sensors, connected to a LoRaWAN Gateway, were used to measure air temperature, RH, CO_2_, and biogenic VOCs in 15-minute intervals [26]. Teams of 2–3 trained researchers were responsible for the installation/removal of the sensors based on standard operating procedures (SOPs).

### 2.5 Questionnaires

Two questionnaires - based on the questionnaires/checklists used in the SINPHONIE project [23] and developed using the REDCap software [24] - were used to collect information about school building/classroom characteristics that potentially have an impact on indoor air quality. Specifically, information was collected about: i) the school characteristics, i.e., year of construction, floor levels, number of classrooms used by children, number of portable–metallic classrooms, whether the school building/classrooms had been recently painted (previous semester), frequency of cleaning activities in classrooms, use of pesticides; and ii) the sampled classrooms’ characteristics and occupancy status during the sampling days, i.e., number of children in the specific classroom, classroom surface area, ventilation system type, windows type and number, doors number, use of air-condition/fans, time periods during which windows/doors/air–condition/fans were open/on. The questionnaires were answered by the school headmaster (beginning of the study) and teachers (at the end of each sampling day), respectively.

### 2.6 Data processing and analysis

A specific data analysis workflow was implemented to process data collected from the sensors and other exposure assessment tools, including the questionnaires (Figure S1).

We estimated descriptive statistics, e.g., mean, median, interquartile range, minimum and maximum per parameter, location (indoors vs outdoors), district and urbanization degree, for the school period (7:45 am–1:05 pm) and the complete sampling period. Time–series plots were created for the visualization of the raw and school–period averaged data per school. Complementary information collected with questionnaires, e.g., timeframe during which windows were open, breaks vs class times or indoors/outdoors was indicated in the plots.

To explore the differences between the indoor and outdoor environmental parameters, we used linear mixed–effect models accounting for the multilevel design (school/classroom) using school–period averaged data for typical classrooms. The geographical district and degree of urbanization were initially used in the random effect part of the model, however they were dropped after checking the Akaike information criterion (AIC) in models with and without these parameters (AIC was similar in both types of models). Outcomes were the indoor parameter levels and predictors included outdoor levels of the same parameter, school period type (breaks with 10-20-min duration vs class periods with 40-min duration), progressing school period in the day [1–10, including class periods (7) and breaks (3)], percentage of open windows (0–100%), open doors (0–100%) and fans in use (0–100%), recent (previous semester) painting of classrooms (yes vs. no) and chlorine use frequency during classroom cleaning (five times a week vs less or equal to three times a week). In models where PM parameters were the outcomes, there was adjustment for indoor air temperature and RH, while in models where air temperature was the outcome, there was adjustment for indoor RH and in models where RH was the outcome, there was adjustment for indoor air temperature. The school–period averages for CO_2_ and VOCs were log–transformed before their use in models to resolve rescaling warnings presented when parameters were used without transformation (raw) in the models.

In a sensitivity analysis, we used linear mixed–effect regression models to explore the differences between the indoor and outdoor environmental parameters only in atypical classes.

Data points of parameters measured with the sensors during school class hours within classrooms were categorized as below, within and/or above cut offs following recommendations or guidelines by European or international organizations. Recommendations used for the different IAQ parameters measured were: (a) 22–27 °C for air temperature in school classrooms in summer based on the EU Standard EN 16798–1:2019 [27], (b) 40–60% for RH in schools based on ASHRAE guidelines [28] (c) 800–1350 ppm for CO_2_ in school classrooms based on the EU Standard EN 16798–1:2019 [27], and (d) 15 μg/m^3^ for PM_2.5_ (24h), and 45 μg/m^3^ for PM_10_ (24h), based on the WHO Air Quality Guidelines [29].

All analyses were performed in R (v.4.1.2) with RStudio (2022.02.0+443).

## 3 Results

### 3.1 Characteristics of schools and classrooms

Overall, 42 public primary schools were included. Sampling took place in June 2021 for 55% of schools and in May 2021 for 41% of schools with the sampling period being one day (24-h) in 55% of schools and two days for the rest schools (Table 1). The selected schools' distribution in the five districts was based on the actual schools' number per district, with 36% in Nicosia, 29% in Limassol, 19% in Larnaca, 12% in Pafos and 5% in Famagusta. The mean year of schools’ construction was in 1963 and the mean number of classrooms per school was 20. The majority of schools had two floors and had not been recently painted (last semester). More than half of headmasters reported that pesticides are used for pest control in their school with a yearly frequency for 39% of schools and less frequent use for 26% of schools.

**Table 1:** Characteristics of the STEPS participating schools and their classrooms.

	Overall
# schools	42
**District (%)**	
Famagusta	2 (4.8)
Larnaca	8 (19.0)
Limassol	12 (28.6)
Nicosia	15 (35.7)
Pafos	5 (11.9)
**Year of school construction (mean (SD))**	1963 (32.87)
**School Floors (%)**	
2	34 (81.0)
1	8 (19.0)
**Number of classes per school (mean (SD))**	19.88 (6.51)
**Number of portable–metallic classrooms (mean (SD))**	0.40 (0.83)
**School painted last semester (%)**	
Yes	11 (26.2)
No	31 (73.8)
**Locations painted (%)**	
Indoors and Outdoors	6 (54.5)
Indoors	2 (18.2)
Outdoors	3 (27.3)
**Pesticides use (%)**	
Yes	23 (54.8)
No	12 (28.6)
Don't know	7 (16.7)
**Pesticides use frequency (%)**	
Every month	3 (13.0)
Every 3 months	1 (4.3)
Every 6 months	4 (14.4)
Every year	9 (39.1)
Less than once a year	6 (26.1)
**Pesticides use location (%)**	
Indoors	1 (4.3)
Indoors and Outdoors	10 (43.5)
Indoor areas (not classrooms)	3 (13.0)
Outdoors	7 (30.4)
Outdoors and indoor areas (not classrooms)	2 (8.7)
**Classrooms' cleaning frequency (%)**	
All school days	42 (100.0)
**Classrooms' cleaning frequency on a daily basis (%)**	
Afternoon	33 (78.6)
Morning and afternoon	2 (4.8)
Morning, afternoon and breaks	3 (7.1)
Morning and breaks	4 (9.5)
**Chlorine use during classrooms cleaning (%)**	
Yes	34 (81.0)
No	8 (19.0)
**Chlorine use frequency during classrooms cleaning (%)**	
Five times a week	26 (76.5)
Three times per week	2 (5.9)
Twice a week	3 (8.8)
Oncea week	3 (8.8)
**Sampling days (%)**	
1	23 (54.8)
2	19 (45.2)
**Sampling month (%)**	
July	1 (2.4)
June	23 (54.8)
May	17 (40.5)
May and June	1 (2.4)

Abiding by the COVID–19 protocol guidelines, all classrooms (100%) of participating schools were daily cleaned, with afternoon being the most usual time of day for cleaning/disinfecting (79%) (Table 1); chlorine was used in 81% of them for disinfection purposes during cleaning (77% daily chlorine disinfection). The mean classroom area was 50 m^2^ and all classrooms could support natural ventilation (windows) (Table S1). The mean number of children in the classrooms was similar for both sampling days (17–18 children). A total of 13 out of the 84 sampled classrooms (15%) were characterized by teachers/headmasters as atypical, i.e., having reported issues, such as, COVID–19 case(s) (85%), dust (8%), or high air temperatures (8%) (Table S2). Similar characteristics were observed between typical and atypical classes (Table S3).

### 3.2 Natural and mechanical ventilation in classrooms

The mean number of windows per classroom was eight and about half of the classrooms had two doors (Table S1). Fan(s) was available in 90% of classrooms and air-conditioning (A/C) in 11% of classrooms. Considering all classrooms, the average number of open windows, doors and A/C in use was the same for all school periods, being open or in use from 7:45 am (Table S4). The median number of fans in use showed an increase after the 4^th^ school period and was not in use during the three school breaks (school periods 3, 6 and 9). The mean (sd) number of open windows in all school periods were 4.1 (2.0) (Table S4). The mean (sd) number of classroom A/C in use was nearly nonexistent [0.1 (0)], while A/C was turned off during school breaks.

### 3.3 Descriptives of air quality parameters

The descriptives of the parameters measured in schools per location during school class hours (7:45 am–1:05 pm) can be found in Table 2. As expected, the mean CO_2_ levels were higher indoors compared to outdoors (518 ppm vs. 441 ppm) and the maximum value was more than double indoors compared to outdoors (1828 ppm vs. 747 ppm). For PM parameters (PM_1,_ PM_2.5_ and PM_10_), slightly higher median values were observed for indoors compared to outdoor locations (Table 2). Mean RH levels were higher indoors compared to outdoors (46% vs. 41%) with the maximum value reaching 75% for indoors and 70% for outdoors. The mean air temperature was about 2 °C higher outdoors compared to indoors with the maximum air temperature indoors and outdoors reaching 43.0 °C and 44.5 °C, respectively. The median VOCs levels were slightly higher indoors compared to outdoors (1125 ppb vs. 1084 ppb). The summary of the parameters measured in schools per location and district during the school class hours and during the whole sampling period can be found in the Supplementary (Tables S5 and S6, respectively). Comparing the school-period averaged indoor values of the parameters between typical and atypical classrooms, during the school class hours (7:45 am–1:05 pm), the mean RH and VOCs were higher in atypical compared to typical classrooms while the mean air temperature was higher in typical compared to atypical classrooms (Table S7).

**Table 2:** Summary of environmental parameters during school class hours in the STEPS classrooms and proximal outdoor school locations.

Parameter	Location	n	Mean	SD	Min	p25	Median	p75	Max
CO_2_ (ppm)	indoors	2450	518	129.6	303	442	485	565.8	1828
CO_2_ (ppm)	outdoors	1491	440.5	69.1	347	401	416	457	747
PM_1_ (μg/m^3^)	indoors	17279	10.9	4.8	0.2	7.3	10.2	13.6	42.5
PM_1_ (μg/m^3^)	outdoors	8828	10.6	4.7	1.6	6.9	9.8	13.2	27.7
PM_10_ (μg/m^3^)	indoors	17279	16.1	6.9	1.4	11	15.1	19.8	52.3
PM_10_ (μg/m^3^)	outdoors	8828	16	7.2	4	10.7	14.6	19.7	47.4
PM_2.5_ (μg/m^3^)	indoors	17279	14.6	6.7	0.7	9.7	13.8	18.1	98.8
PM_2.5_ (μg/m^3^)	outdoors	8828	14.3	6.4	2.9	9.4	13.1	17.7	41.6
RH (%)	indoors	2450	46.4	9.7	21	39	46.0	54.5	75
RH (%)	outdoors	1491	41.4	10.2	16.5	34.5	40.5	48.5	69.5
Air temp. (°C)	indoors	2450	29.3	2.5	25.3	27.5	28.9	30.3	42.9
Air temp. (°C)	outdoors	1491	31.1	3.4	24.9	28.9	30.4	32.7	44.5
VOCs (ppb)	indoors	2450	3333.5	8529.4	499	658	1125	2114.8	65535
VOCs (ppb)	outdoors	1491	9331.5	18268.5	499	594.5	1084	4815.5	65535

SD: standard deviation, p25: 25^th^ percentile, p75: 75^th^ percentile.

### 3.4 Temporal dynamics of IAQ

**Figure 1:**
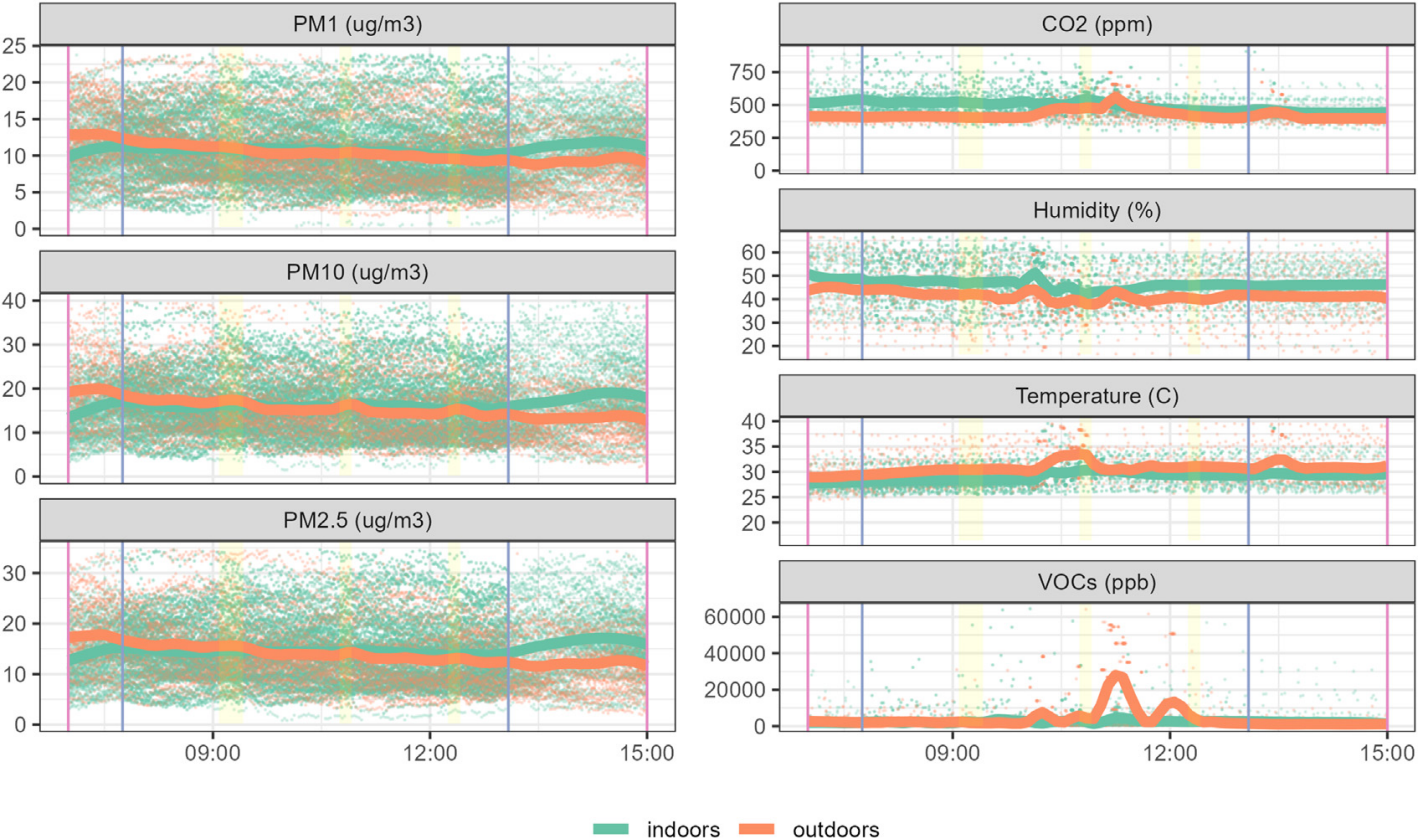
Time–series plots of environmental parameters (raw values – 99^th^ percentile) from all 42 schools including data points from 7:00 am to 3:00 pm, without taking in account the sampling date. Purple lines indicate school opening and closing times (7:00 am and 3:00 pm), blue lines indicate the classes start and end times (7:45 am and 1:05 pm) and yellow shadowed areas indicate the school break times (9:00–9:25 am, 10:45–10:55 am and 12:15–12:25 pm). Green and orange lines are smooth lines based on LOESS curve fitting denoting average trends across all schools (green for indoors, orange for outdoors).

Plotting only the school opening–closing hours (opening doors at 7 am until closing of doors at 3 pm), indoor air temperature's temporal dynamics followed similar pattern to that of outdoor air temperature, exhibiting an increasing trend over time and with a spike at about 10:30 am (Figure 1). The typical inverse association between indoor temperature and RH was depicted in the temporal patterns of indoor RH in classrooms. The indoor CO_2_ levels increased before the classes began and started decreasing thereafter. In general, the indoor and outdoor VOCs levels were relatively stable with the exception of spikes before and after the second school break. The indoor PM levels were lower than their respective outdoor levels at 7am when doors opened, but they quickly equilibrated by the time the class began and remained more or less stable thereafter until the end of classes (Figure 1). Following the end of classes and until the school closing time there was a further increase in the PM levels.

The time–series plots showing the variance of the parameters indoors and outdoors of classrooms throughout the sampling period (May 12 – July 7, 2021) can be found in Figure S2.

### 3.5 Differences between indoor and outdoor air quality parameters

The indoor levels of all parameters were positively associated (p < 0.001) with their corresponding outdoor levels (Tables 3 and 4). Indoor PM, air temperature, RH and VOCs were positively associated with the school period, while indoor CO_2_ was negatively associated with the school period (β = -0.016, 95% CI: -0.019–-0.013, p < 0.001) (Tables 3 and 4). Indoor CO_2_ was negatively associated with the percentage of open windows (β = -0.001, 95% CI: -0.001–0.000, p < 0.01, Table 4) while the indoor PM levels were positively associated with the percentage of open windows (β = 0.024, 95% CI: 0.019–0.029, p < 0.001 for PM_2.5_, Table 3).

**Table 3:** Linear mixed effect models of PM parameters measured in typical school classrooms (indoors) during school class hours regressed on the outdoor levels and adjusted for indoor air temperature and RH levels, period type (break vs class time), school period (1–10), percentage of open windows, open doors and fans in use, recent paint inside the classroom (yes vs no) and chlorine use frequency during classrooms’ cleaning (five times per week vs less or equal to three times per week).

Predictors	Indoor PM_1_	Indoor PM_2.5_	Indoor PM_10_
	Estimate (95% CI)	Estimate (95% CI)	Estimate (95% CI)
Outdoor PM_1_	0.804 ∗∗∗ (0.791–0.817)		
Indoor air temperature	0.080 ∗∗ (0.022–0.139)	0.084 (-0.006 ​– ​0.173)	0.090 (-0.017 ​– ​0.197)
Indoor RH	-0.025 ∗∗∗ (-0.033 ​– ​-0.018)	-0.040 ∗∗∗ (-0.051 ​– ​-0.028)	-0.030 ∗∗∗ (-0.044 ​– ​-0.016)
School break	0.049 (-0.024 ​– ​0.122)	0.042 (-0.071 ​– ​0.154)	0.045 (-0.089 ​– ​0.179)
School period	0.014 ∗ (0.000–0.028)	0.032 ∗∗ (0.011–0.054)	0.046 ∗∗∗ (0.020–0.072)
% open windows	0.015 ∗∗∗ (0.012–0.018)	0.024 ∗∗∗ (0.019–0.029)	0.027 ∗∗∗ (0.021–0.032)
% fans in use	-0.000 (-0.001 ​– ​0.001)	0.004 ∗∗∗ (0.002–0.005)	0.005 ∗∗∗ (0.003–0.007)
% open doors	-0.002 ∗ (-0.004 ​– ​-0.000)	-0.002 (-0.006 ​– ​0.001)	-0.001 (-0.005 ​– ​0.003)
Recent painting of classroom	-0.611 (-2.180 ​– ​0.959)	-1.515 (-3.786 ​– ​0.755)	-1.464 (-4.088 ​– ​1.161)
Five times a week use of chlorine during classroom cleaning	0.270 (-1.007 ​– ​1.546)	0.392 (-1.454 ​– ​2.239)	0.771 (-1.365 ​– ​2.906)
Outdoor PM_2.5_		0.743 ∗∗∗ (0.728–0.757)	
Outdoor PM_10_			0.719 ∗∗∗ (0.703–0.735)
Residual variance	1.35	3.25	4.62
Classroom-level random intercept variance	3.37	7.04	9.41
ICC	0.71	0.68	0.67
Number of schools	32	32	32
Number of classrooms	2	2	2
Number of measurements	8992	9105	9072

Models' details:

(a) Environmental parameters (indoors and outdoors) are school-period averages.

(b) Random intercepts for the repeated measurements within classrooms, and classrooms nested within schools, with unstructured covariance matrix.

(c) ∗*p < 0.05 ∗∗p < 0.01 ∗∗∗p < 0.001*.

Abbreviations: PM: particulate matter, RH: relative humidity, ICC: intraclass correlation coefficient.

**Table 4:** Linear mixed effect models of indoor air quality for MCF parameters measured in typical school classrooms (indoors) during school class hours regressed on the outdoor levels and adjusted for period type (break vs class time), school period (1–10), percentage of open windows, open doors and fans in use, recent paint inside the classroom (yes vs no) and chlorine use frequency during classrooms’ cleaning (five times per week vs less or equal to three times per week).

Predictors	Indoor air temperature	Indoor RH	Indoor CO_2_	Indoor VOCs
	Estimate (95% CI)	Estimate (95% CI)	Estimate (95% CI)	Estimate (95% CI)
Outdoor air temperature	0.140 ∗∗∗ (0.116–0.164)			
Indoor RH	-0.013 ∗∗∗ (-0.019 ​– ​-0.007)			
School break	0.113 ∗∗∗ (0.049–0.178)	-0.819 ∗∗∗ (-1.168 ​– ​-0.470)	0.006 (-0.014 ​– ​0.026)	-0.001 (-0.141 ​– ​0.139)
School period	0.104 ∗∗∗ (0.093–0.116)	0.350 ∗∗∗ (0.285–0.415)	-0.016 ∗∗∗ (-0.019 ​– ​-0.013)	0.036 ∗∗ (0.014–0.058)
% open windows	-0.001 (-0.002 ​– ​0.001)	-0.010 (-0.020 ​– ​0.000)	-0.001 ∗∗ (-0.001 ​– ​-0.000)	-0.002 (-0.005 ​– ​0.002)
% fans in use	0.000 (-0.001 ​– ​0.001)	0.001 (-0.004 ​– ​0.006)	0.000 (-0.000 ​– ​0.000)	0.001 (-0.000 ​– ​0.003)
% open doors	-0.001 (-0.003 ​– ​0.001)	0.010 ∗ (0.000–0.019)	-0.000 (-0.001 ​– ​0.000)	-0.000 (-0.003 ​– ​0.003)
Recent painting of classroom	-0.125 (-1.293 ​– ​1.043)	-5.265 ∗∗ (-9.161 ​– ​-1.369)	0.038 (-0.074 ​– ​0.150)	-0.238 (-0.689 ​– ​0.213)
Five times a week use of chlorine during classroom cleaning	1.425 ∗∗ (0.419–2.430)	-0.970 (-4.360 ​– ​2.419)	0.104 ∗ (0.007–0.201)	0.191 (-0.212 ​– ​0.593)
Outdoor RH		0.615 ∗∗∗ (0.591–0.639)		
Indoor air temperature		-1.244 ∗∗∗ (-1.489 ​– ​-0.999)		
Outdoor CO_2_			0.675 ∗∗∗ (0.514–0.836)	
Outdoor VOCs				0.201 ∗∗∗ (0.135–0.268)

Residual variance	0.18	5.45	0.01	0.48
Classroom-level random intercept variance	2.19	24.31	0.02	0.30
ICC	0.92	0.82	0.67	0.38
Number of schools	34	34	34	34
Number of classrooms	2	2	2	2
Number of measurements	1598	1598	866	866

Models' details:

(a) Environmental parameters (indoors and outdoors) are school-period averages.

(b) CO_2_ and VOCs (indoors and outdoors) are log-transformed.

(c) Random intercepts for the repeated measurements within classrooms, and classrooms nested within schools, with unstructured covariance matrix.

(d) *∗p < 0.05 ∗∗p < 0.01 ∗∗∗p < 0.001*.

Abbreviations: PM: particulate matter, RH: relative humidity, CO_2_: carbon dioxide, VOCs: volatile organic compounds, ICC: intraclass correlation coefficient.

The indoor PM levels were negatively associated with indoor RH (e.g., β = -0.040, 95% CI: -0.051–-0.028, p < 0.001 for PM_2.5_) (Table 3). The indoor PM_1_ levels were positively associated with indoor air temperature (β = 0.080, 95% CI: 0.022–0.139, p < 0.01) and the indoor PM_2.5_ and PM_10_ were positively associated with the percentage of fans in use (β = 0.004, 95% CI: 0.002–0.005, p < 0.001 and β = 0.005, 95% CI: 0.003–0.007, p < 0.001, respectively). Indoor RH was positively associated with the percentage of open doors (β = 0.010, 95% CI: 0.000–0.019, p < 0.05, Table 4) while indoor PM_1_ was negatively associated with the percentage of open doors (β = -0.002, 95% CI: -0.004–0.000, p < 0.05, Table 3).

Indoor air temperature was positively associated with the frequent – five times a week – use of chlorine during classroom cleaning (β = 1.425, 95% CI: 0.419–2.430, p < 0.01) and the school break (β = 0.073, 95% CI: 0.008–0.138, p < 0.001), while indoor RH was negatively associated with the school break (β = -0.819, 95% CI: -1.168–-0.470, p < 0.001) and the recent painting of classrooms (β = -5.265, 95% CI: -9.161–-1.369, p < 0.01) (Table 4).

Sensitivity analysis using only data from atypical classes, showed some differences (Tables S8 and S9). In atypical classes, the indoor PM levels were negatively associated with the percentage of open windows (e.g., β = -0.032, 95% CI: -0.037–-0.027, p < 0.001 for PM_2.5_) and the school break (e.g., β = -0.526, 95% CI: -0.634–-0.418, p < 0.001 for PM_2.5_) (Table S8). Indoor air temperature and RH were positively associated with the percentage of open windows (β = 0.008, 95% CI: 0.006–0.011, <0.001, and β = 0.041, 95% CI: 0.020–0.062, p < 0.001, respectively) and the percentage of fans in use (β = 0.003, 95% CI: 0.002–0.004, p < 0.001, and β = 0.019, 95% CI: 0.011–0.028, <0.001, respectively) (Table S9).

### 3.6 Comparisons with recommendations/guidelines of international organizations

Most of the school time (85%), indoor air temperature was consistently higher than the upper limit of 27 °C recommended by the European standard on the energy performance of buildings (Table 5) [27]. This was corroborated by the observation that only a quarter of the air temperature values were below 27.5 °C (Table 2). More than half of the school time, the indoor RH was within the recommended range of 40–60% (Table 5) [28], whereas more than a quarter of the time, indoor RH was below 40% and 8% of RH measurements were >60%. Almost all indoor CO_2_ measurements (98%) were within the recommended guidelines (≤800 ppm) [27] (Table 5) and more than three quarters of the values were below 566 ppm (Table 2). All 24-hour PM_10_ values were within the WHO air quality guidelines [29] (≤45 μg/m^3^, Table 5), with more than three quarters of the raw values during school class hours being below 20 μg/m^3^ (Table 2). However, about one third of the 24-hour indoor PM_2.5_ values (33%) exceeded the guideline value of 15 μg/m^3^ [29], and about half of the raw values during school class hours were higher than 14 μg/m^3^ (Table 2). Percentage of time exceedances for the above-mentioned parameters varied between geographical districts and degree of urbanization areas where schools were located (Table S10).

**Table 5:** Percent exceedances of select indoor air quality indicators during school class hours, based on international cutoffs. For PM_2.5_ and PM_10_, 24–h data were used.

Parameter	Total n. values	Categories	n. values per category	% values per category
Air temperature	2450	>27 °C†	2080	85
Air temperature	2450	22°C–27 °C†	370	15
RH	2450	<40%‡	684	28
RH	2450	>60%‡	187	8
RH	2450	40%–60%‡	1579	64
CO_2_	2450	≤800 ppm†	2390	98
CO_2_	2450	>1350 ppm†	11	0
CO_2_	2450	800–1350 ppm†	49	2
PM_2.5_	82	≤15 μg/m^3^§	55	67
PM_2.5_	82	>15 μg/m^3^§	27	33
PM_10_	82	≤45 μg/m^3^§	82	100

† Based on the EU Standard EN 16798-1:2019 Energy Performance of Buildings [27].

‡ Based on the ASHRAE guidelines [28].

§ Based on the WHO Air Quality Guidelines [29].

## 4 Discussion

We designed and conducted a school population representative, cross sectional study to describe the magnitude and variance of IAQ parameters for 42 public primary schools located in densely and intermediately populated areas in Cyprus, during May–July 2021. Using a suite of tools, ranging from questionnaires and diaries to stationary sensors, we observed that the median values of CO_2_, PM_1_, PM_2.5_, PM_10_, RH and VOCs in typical classrooms were higher indoors than outdoors; the opposite trend was observed for air temperature. The indoor levels of all parameters measured were positively associated with their corresponding outdoor levels. The percentage of open windows was positively associated with the indoor PM levels and negatively associated with indoor CO_2_. As the school periods progressed, the levels of indoor PM, air temperature, RH and VOCs showed an increase while indoor CO_2_ decreased. The recommended EU cut–off for indoor air temperature during summer's period of the study (May–July) was not respected in 85% of school time, while 33% of the 24-hour PM_2.5_ measurements exceeded the WHO-recommended guideline value.

Compared to the EU–wide SINPHONIE study results covering indoor air pollutants, thermal and physical parameters from 115 schools and 23 European countries [11], lower indoor median PM_2.5_ (14 μg/m^3^ vs. 38 μg/m^3^) and CO_2_ (485 ppm vs. 1370 ppm), but higher median air temperature (29 °C vs. 22 °C) and RH (46% vs. 40%) were observed in our study. However, the results are not directly comparable between the two studies as they were conducted in two different seasons (SINPHONIE took place in winter-spring, November 2011 to March 2012 vs. our study during May to July 2021). A French study in eight schools during winter measured the indoor and outdoor concentrations of nitrogen oxides, ozone and particles showing the impact of outdoor contaminants on indoor exposures [30]. Similarly, PM_2.5_, NO_2_, and ultra-fine particles measured in 39 schools in Barcelona in both indoor and outdoor environments were higher than expected [31].

The fact that our study took place during May–July makes it unique, as the majority of published school IAQ studies did not take place in that period when heat stress might be more noticeable. A U.S. study across 16 schools in the mid-Atlantic region from December 2015 to May 2017 investigated the association of infrastructural and contextual conditions with IAQ features and showed that seasonality and microclimatic parameters (temperature, relative humidity and ventilation) were important factors with significant impacts on indoor exposure levels [32].

In our study, windows and doors of the classrooms were open from the beginning of the school day, while classroom cleaning/disinfection was daily practiced in most school classrooms, abiding by the COVID-19 school protocol in place. Indeed, the CO_2_ levels in the classrooms tended to be well within the internationally acceptable limits, suggesting adequate ventilation for the participating schools. This is a key IAQ indicator of ventilation with consequences for microbiological spread and control. A Portuguese study in 73 classrooms monitored in 20 public primary schools (November–March) highlighted the IAQ problems in poorly-ventilated classrooms where various pollutant sources exist [33]. A study in south Italy involving 12 lower secondary schools in Gela industrial area showed poor ventilation and high concentration levels of CO_2_ and NO_2_ under occupancy [34].

Our study has several strengths. We measured various air quality parameters, indoors and outdoors, in a school population representative sample randomly selected from the list of eligible schools all over Cyprus located in areas classified with degree of urbanization 1 or 2 (42 schools: 84 classrooms and 42 outdoor locations). The studied classrooms and schools exhibited variability in terms of surface area, construction age, number of windows and availability of fans or A/C in each classroom, and this provided a more comprehensive assessment of school environments.

Limitations of our study included the fact that the sampling period was shorter in some schools compared to others (i.e., 24-h sampling for 54% of schools), due to time restrictions; however, a larger number of schools than what was originally estimated were included in the study, hence, further increasing the power of the study. Monitoring during all seasons is essential to evaluate seasonal effects, as different conditions may apply in winter, such as, fewer open windows/doors/fans may be in use. Location of schools (e.g., proximity to traffic) could be taken into account for better assessment of PM and VOCs variability.

Clean air for all is a human right and an important European policy goal. Within the school environment, multiple exposure agents and sources may co-exist, which when coupled with lifestyle/behavioral choices, building/surfaces characteristics and relevant policies or programs, such as the COVID-19 school protocols or climate adaptation schemes comprise a dynamic exposome profile implicated with a series of communicable [35] (e.g., COVID-19) or non-communicable diseases (e.g., lung function impairment, asthma, allergies). However, there is so far limited evidence from indoor-based epidemiological studies of children's health.

The interlinkages between children's lifestyles and behaviors and the dynamically evolving indoor air exposome are warranted to be studied in detail, if we were to better understand the association between IAQ and its attributable burden of disease. Integrating knowledge of the indoor air exposome in schools should be leveraged to test and apply innovative technologies for monitoring and improving IAQ. This can further foster a science-based framework, for public and private actors, to consider IAQ issues together with climate adaptation strategies, and economic, environmental, and social dimensions, in their policy setting and investment decisions. Further emphasis on IAQ dynamics in schools is needed to strengthen the adaptive capacity and resilience of EU populations and health systems to climate change and its manifestations as they disproportionally affect vulnerable populations, like children.

## 5 Conclusions

The STEPS provided us with an in-depth analysis of the IAQ status in primary schools of Cyprus, when the May–July 2021 COVID-19 school protocol measures were in place. Using a suite of exposomic tools, the STEPS monitored and analyzed physical (air temperature, RH), particulates (various size fractions of PM) and chemical pollutants (CO_2_, VOCs) using low-cost sensors and questionnaire-based information on each participating school/classroom during the Cyprus-wide 2021 COVID-19 school protocol implementation. Most of the school time (85%), the indoor air temperature was higher than the upper limit of 27 °C recommended by the European standard on the energy performance of buildings, while 33% of the 24-hour PM_2.5_ measurements exceeded the WHO-recommended guideline value. Natural ventilation in classrooms with windows and doors being systematically open in practice showed that the health protocol in place was respected and helped in keeping CO_2_ levels within acceptable levels for the majority of school time. During the COVID-19 pandemic era, it is important to align health policies and programs for schools with monitoring their compliance in practice and their apparent effectiveness in controlling key metrics of environment and health outcomes.

The deployment of IAQ monitoring schemes within a broader methodological framework of the indoor exposome in schools would pave the way for quantifying the cost of the attributable burden of disease, and thus monetizing possible IAQ and health interventions in a changing climate. More environmental and health data is needed to support the new WHO Global Air Quality guidelines that were recently put forward (September 2021). Such efforts would further strengthen health arguments for climate action in school systems, as well as in other indoor settings.

## Declarations

### Author contribution statement

Corina Konstantinou: Performed the experiments; Analyzed and interpreted the data; Wrote the paper.

Andria Constantinou & Eleni G. Kleovoulou: Performed the experiments; Wrote the paper.

Alexis Kyriacou, George Millis & Konstantinos C. Makris: Conceived and designed the experiments; Performed the experiments; Analyzed and interpreted the data; Contributed reagents, materials, analysis tools or data; Wrote the paper.

Christina Kakoulli: Analyzed and interpreted the data; Wrote the paper.

Michalis Michaelides: Conceived and designed the experiments; Performed the experiments; Analyzed and interpreted the data; Wrote the paper.

### Funding statement

K.C. Makris was supported by the European Union's Horizon 2020 research and innovation programme (EXPOSOGAS project) under grant agreement (810995).

A. Kyriacou would like to acknowledge funding from the European Union and the Republic of Cyprus through the Research and Innovation Foundation (PRE-SEED/0719/0148 and SEED COVID/0420/0026).

G. Milis was supported by Cyprus Research and Innovation Foundation (Programme RESTART 2016–2020/I. SMART GROWTH/RESEARCH IN ENTERPRISES/ENTERPRISES/0618/0132, Domognostics+).

### Data availability statement

Data, scripts and outputs are available here: DOI: https://doi.org/10.5281/zenodo.6520242.

### Declaration of interests statement

The authors declare no conflict of interest.

### Additional information

No additional information is available for this paper.

## Acknowledgements

K.C. Makris would like to acknowledge partial funding from the European Union’s Horizon 2020 research and innovation programme under grant agreement #810995 (EXPOSOGAS project). We would like to thank Dr. Xanthi Andrianou for her contributions in the methodology and formal data analysis. A. Kyriacou would like to acknowledge funding from the European Union and the Republic of Cyprus through the Research and Innovation Foundation (PRE-SEED/0719/0148 and SEED-COVID/0420/0026). G. Milis would like to acknowledge funding from the Cyprus Research Promotion Foundation / Programme RESTART 2016-2020 / I. SMART GROWTH / RESEARCH IN ENTERPRISES/ ENTERPRISES/0618/0132, Domognostics+. Furthermore, we would like to thank the Cyprus Ministry of Education and headmasters for providing access to the school premises during the COVID-19 pandemic period.

## Appendix A Supplementary data

Supplementary-Tables and Figures_final_ER Supplementary-Tables and Figures_final_ER
