# Supplementary material for: Assessment of indoor and outdoor air quality in primary schools of Cyprus during the COVID–19 pandemic measures in May–July 2021
Source: Heliyon. 2022 May 2;8(5):e09354. doi: 10.1016/j.heliyon.2022.e09354 (PMC9057936; doi:10.1016/j.heliyon.2022.e09354)
Supplement: Supplementary-Tables and Figures_final_ER [file mmc1.docx]

**Supporting Information**

**SI1**

Assessment of indoor and outdoor air quality in primary schools of Cyprus during the COVID-19 pandemic measures in May-July 2021

Corina Konstantinou^1^, Andria Constantinou^1^, Eleni G. Kleovoulou^1^, Alexis Kyriacou^2^, Christina Kakoulli^4^, George Millis^3^, Michalis Michaelides^4^, Konstantinos C. Makris^1*^

^1^Cyprus International Institute for Environmental and Public Health (CII), Cyprus University of Technology, Limassol, Cyprus

^2^LELANTUS Innovations Ltd, Nicosia, Cyprus

^3^PHOEBE Research & Innovation Ltd, Nicosia, Cyprus

^4^Department of Electrical Engineering, Computer Engineering and Informatics, Cyprus University of Technology, Limassol, Cyprus

# **Tables**

Table S1 Classrooms’ characteristics. Two classrooms in each school of the STEPS study.

|  | **Overall** | **Classroom 1** | **Classroom 2** |
| --- | --- | --- | --- |
| n | 84 | 42 | 42 |
| **Classroom area in m^2^ (mean (SD))** | 50.27 (11.84) | 50.46 (10.91) | 50.08 (12.84) |
| **Ventilation type (%)** |  |  |  |
| Natural | 81 (96.4) | 40 (95.2) | 41 (97.6) |
| Natural and automatic | 3 (3.6) | 2 (4.8) | 1 (2.4) |
| **Number of windows (mean (SD))** | 7.61 (3.29) | 7.57 (3.47) | 7.64 (3.14) |
| **Number of doors (%)** |  |  |  |
| 2 | 46 (54.8) | 21 (50.0) | 25 (59.5) |
| 1 | 34 (45.2) | 21 (50.0) | 17 (40.5) |
| **Air condition (A/C) / Fan available in classroom (%)** |  |  |  |
| A/C | 2 (2.4) | 1 (2.4) | 1 (2.4) |
| Fan | 69 (82.1) | 34 (81.0) | 35 (83.3) |
| Fan and A/C | 7 (8.3) | 4 (9.5) | 3 (7.1) |
| None | 6 (7.1) | 3 (7.1) | 3 (7.1) |
| **Number of fans (mean (SD))** | 2.14 (0.39) | 2.13 (0.41) | 2.16 (0.37) |
| **Number of children - first sampling day (mean (SD))** | 17.56 (4.02) | 17.19 (4.42) | 17.93 (3.58) |
| **Air condition on - first sampling day (%)** |  |  |  |
| Yes | 4 (44.4) | 2 (40.0) | 2 (50.0) |
| No | 5 (55.6) | 3 (60.0) | 2 (50.0) |
| **Fan no. 1 on - first sampling day (%**) |  |  |  |
| Yes | 53 (69.7) | 27 (71.1) | 26 (68.4) |
| No | 23 (30.3) | 11 (28.9) | 12 (31.6) |
| **Fan no. 2 on - first sampling day (%**) |  |  |  |
| Yes | 46 (61.3) | 24 (64.9) | 22 (57.9) |
| No | 29 (38.7) | 10 (35.1) | 16 (42.1) |
| **Fan no. 3 on - first sampling day (%**) |  |  |  |
| Yes | 5 (41.7) | 3 (50.0) | 2 (33.3) |
| No | 7 (30.3) | 3 (50.0) | 4 (66.7) |
| **Number of children - 2^nd^ sampling day (mean (SD))** | 17.29 (3.57) | 17.58 (4.40) | 17.00 (2.58) |
| **Air condition on - 2^nd^ sampling day (%)** |  |  |  |
| Yes | 4 (50.0) | 2 (40.0) | 2 (66.7) |
| No | 4 (50.0) | 3 (60.0) | 1 (33.3) |
| **Fan no. 1 on - 2^nd^ sampling day (%**) |  |  |  |
| Yes | 23 (65.7) | 11 (64.7) | 12 (66.7) |
| No | 12 (34.3) | 6 (35.3) | 6 (33.3) |
| **Fan no. 2 on - 2^nd^ sampling day (%**) |  |  |  |
| Yes | 20 (55.6) | 10 (55.6) | 10 (55.6) |
| No | 16 (44.4) | 8 (44.4) | 8 (44.4) |
| **Fan no. 3 on - 2^nd^ sampling day (%**) |  |  |  |
| Yes | 4 (66.7) | 2 (66.7) | 2 (66.7) |
| No | 2 (33.3) | 1 (33.3) | 1 (33.3) |

Table S2 Overview of classrooms included in the study (issue refers to the issue reported by teachers/headmasters with regards to this classroom to be defined as “atypical)

| **District** | **Degurba classification** | **Atypical classes** | **Issue** | **n** |
| --- | --- | --- | --- | --- |
| Famagusta | Intermediate density areas |  |  | 2 |
| Larnaka | Densely populated areas | one classroom | COVID-19 | 2 |
| Larnaka | Densely populated areas | one classroom | COVID-19 | 1 |
| Larnaka | Densely populated areas |  |  | 2 |
| Larnaka | Intermediate density areas | both classrooms | COVID-19 | 1 |
| Larnaka | Intermediate density areas |  |  | 2 |
| Limassol | Densely populated areas | one classroom | COVID-19 | 2 |
| Limassol | Densely populated areas | one classroom | Dust | 1 |
| Limassol | Densely populated areas | both classrooms | COVID-19 | 2 |
| Limassol | Densely populated areas |  |  | 5 |
| Limassol | Intermediate density areas |  |  | 2 |
| Nicosia | Densely populated areas | one classroom | High temperature | 1 |
| Nicosia | Densely populated areas |  |  | 12 |
| Nicosia | Intermediate density areas |  |  | 2 |
| Paphos | Intermediate density areas |  |  | 5 |

Table S3 Classrooms’ characteristics ("typical" vs "atypical" - based on teachers' assessment).

|  | **Overall** | **Typical** | **Atypical** |
| --- | --- | --- | --- |
| n | 84 | 71 | 13 |
| **Classroom area in m^2^ (mean (SD))** | 50.27 (11.84) | 50.42 (12.00) | 49.46 (11.38) |
| **Ventilation type (%)** |  |  |  |
| Natural | 81 (96.4) | 68 (95.8) | 13 (100.0) |
| Natural and automatic | 3 (3.6) | 3 (4.2) | 0 (0.0) |
| **Number of windows (mean (SD))** | 7.61 (3.29) | 7.65 (3.38) | 7.38 (2.84) |
| **Number of doors (%)** |  |  |  |
| 2 | 46 (54.8) | 36 (50.7) | 10 (76.9) |
| 1 | 38 (45.2) | 35 (49.3) | 3 (23.1) |
| **Air condition/fan available in classroom (%)** |  |  |  |
| AC | 2 (2.4) | 2 (2.8) | 0 (0.0) |
| Fan | 69 (82.1) | 57 (80.3) | 12 (92.3) |
| Fan and AC | 7 (8.3) | 6 (8.5) | 1 (7.7) |
| None | 6 (7.1) | 6 (8.5) | 0 (0.0) |
| **Number of fans (mean (SD))** | 2.14 (0.39) | 2.16 (0.41) | 2.08 (0.28) |
| **Number of children on first sampling day (mean (SD))** | 17.56 (4.02) | 17.69 (4.06) | 16.85 (3.87) |
| **Air condition on - first sampling day (%)** |  |  |  |
| Yes | 4 (44.4) | 4 (50.0) | 0 (0.0) |
| No | 5 (55.6) | 4 (50.0) | 1 (100.0) |
| **Fan no. 1 on - first sampling day (%**) |  |  |  |
| Yes | 53 (69.7) | 44 (69.8) | 9 (69.2) |
| No | 23 (30.3) | 19 (30.2) | 4 (30.8) |
| **Fan no. 2 on - first sampling day (%**) |  |  |  |
| Yes | 46 (61.3) | 37 (59.7) | 9 (69.2) |
| No | 29 (38.7) | 25 (40.3) | 4 (30.8) |
| **Fan no. 3 on - first sampling day (%**) |  |  |  |
| Yes | 5 (41.7) | 5 (45.5) | 0 (0.0) |
| No | 7 (58.3) | 6 (54.5) | 1 (100.0) |
| **Number of children on 2^nd^ sampling day (mean (SD))** | 17.29 (3.57) | 17.55 (3.51) | 16.14 (3.89) |
| **Air condition on - 2^nd^ sampling day (%)** |  |  |  |
| Yes | 4 (50.0) | 4 (57.1) | 0 (0.0) |
| No | 4 (50.0) | 3 (42.9) | 1 (100.0) |
| **Fan no. 1 on - 2^nd^ sampling day (%**) |  |  |  |
| Yes | 23 (65.7) | 20 (69.0) | 3 (50.0) |
| 1No | 12 (34.3) | (31.0) | 3 (50.0) |
| **Fan no. 2 on - 2^nd^ sampling day (%**) |  |  |  |
| Yes | 20 (55.6) | 17 (58.6) | 3 (42.9) |
| No | 16 (44.4) | 12 (41.4) | 4 (57.1) |
| **Fan no. 3 on - 2^nd^ sampling day (%**) |  |  |  |
| Yes | 4 (66.7) | 4 (80.0) | 0 (0.0) |
| No | 2 (33.3) | 1 (20.0) | 1 (100.0) |

Table S4 Summary table for ventilation parameters (air-condition, doors, fans and windows) at schools during the school periods. School periods 3, 6 and 9 indicate breaks while in the rest periods classes take place. For air-condition and fans: The number denotes how many are on. For doors and windows: The number denotes how many are open.

| **School period** | **Ventilation type** | **n** | **Mean** | **SD** | **Min** | **p25** | **Median** | **p75** | **Max** |
| --- | --- | --- | --- | --- | --- | --- | --- | --- | --- |
| 1 | air-condition | 336 | 0 | 0 | 0 | 0 | 0 | 0 | 1 |
| 2 | air-condition | 336 | 0 | 0 | 0 | 0 | 0 | 0 | 1 |
| 3 | air-condition | 168 | 0 | 0 | 0 | 0 | 0 | 0 | 1 |
| 4 | air-condition | 336 | 0 | 0 | 0 | 0 | 0 | 0 | 1 |
| 5 | air-condition | 336 | 0.1 | 0 | 0 | 0 | 0 | 0 | 1 |
| 6 | air-condition | 168 | 0 | 0 | 0 | 0 | 0 | 0 | 1 |
| 7 | air-condition | 336 | 0.1 | 0 | 0 | 0 | 0 | 0 | 1 |
| 8 | air-condition | 336 | 0.1 | 0 | 0 | 0 | 0 | 0 | 1 |
| 9 | air-condition | 168 | 0 | 0 | 0 | 0 | 0 | 0 | 1 |
| 10 | air-condition | 336 | 0.1 | 0 | 0 | 0 | 0 | 0 | 1 |
| 1 | door | 168 | 1.3 | 1 | 0 | 1 | 1 | 2 | 2 |
| 2 | door | 168 | 1.3 | 1 | 0 | 1 | 1 | 2 | 2 |
| 3 | door | 168 | 1.4 | 1 | 0 | 1 | 1 | 2 | 2 |
| 4 | door | 168 | 1.4 | 1 | 0 | 1 | 1 | 2 | 2 |
| 5 | door | 168 | 1.3 | 1 | 0 | 1 | 1 | 2 | 2 |
| 6 | door | 168 | 1.4 | 1 | 0 | 1 | 1 | 2 | 2 |
| 7 | door | 168 | 1.4 | 1 | 0 | 1 | 1 | 2 | 2 |
| 8 | door | 168 | 1.3 | 1 | 0 | 1 | 1 | 2 | 2 |
| 9 | door | 168 | 1.4 | 1 | 0 | 1 | 1 | 2 | 2 |
| 10 | door | 168 | 1.3 | 1 | 0 | 1 | 1 | 2 | 2 |
| 1 | fan | 336 | 1 | 1 | 0 | 0 | 0 | 2 | 3 |
| 2 | fan | 336 | 0.9 | 1 | 0 | 0 | 0 | 2 | 3 |
| 3 | fan | 168 | 0.7 | 1 | 0 | 0 | 0 | 2 | 3 |
| 4 | fan | 336 | 1.1 | 1 | 0 | 0 | 1 | 2 | 3 |
| 5 | fan | 336 | 1.1 | 1 | 0 | 0 | 1.5 | 2 | 3 |
| 6 | fan | 168 | 0.9 | 1 | 0 | 0 | 0 | 2 | 3 |
| 7 | fan | 336 | 1.2 | 1 | 0 | 0 | 2 | 2 | 3 |
| 8 | fan | 336 | 1.2 | 1 | 0 | 0 | 2 | 2 | 3 |
| 9 | fan | 168 | 0.9 | 1 | 0 | 0 | 0 | 2 | 3 |
| 10 | fan | 336 | 1.2 | 1 | 0 | 0 | 2 | 2 | 3 |
| 1 | window | 168 | 4 | 3 | 0 | 2 | 4 | 5 | 16 |
| 2 | window | 168 | 4.1 | 2 | 0 | 2 | 4 | 5 | 16 |
| 3 | window | 168 | 4.1 | 2 | 0 | 2 | 4 | 5 | 16 |
| 4 | window | 168 | 4.1 | 2 | 0 | 2.2 | 4 | 5 | 16 |
| 5 | window | 168 | 4.1 | 2 | 0 | 3 | 4 | 5 | 16 |
| 6 | window | 168 | 4.1 | 2 | 0 | 3 | 4 | 5 | 16 |
| 7 | window | 168 | 4.1 | 2 | 0 | 3 | 4 | 5 | 16 |
| 8 | window | 168 | 4.1 | 2 | 0 | 3 | 4 | 5 | 16 |
| 9 | window | 168 | 4.1 | 2 | 0 | 3 | 4 | 5 | 16 |
| 10 | window | 168 | 4.1 | 2 | 0 | 3 | 4 | 5 | 16 |

Table S5 Summary table for environmental parameters at schools during the school class period (by district and location)

| **Parameter** | **District** | **Location** | **n** | **Mean** | **SD** | **Min** | **p25** | **Median** | **p75** | **Max** |
| --- | --- | --- | --- | --- | --- | --- | --- | --- | --- | --- |
| CO_2_ (ppm) | Famagusta | indoors | 109 | 482.6 | 95.9 | 398 | 416 | 433 | 533 | 967 |
| CO_2_ (ppm) | Famagusta | outdoors | 82 | 458 | 54.3 | 395 | 411 | 433.5 | 501 | 557 |
| CO_2_ (ppm) | Larnaka | indoors | 544 | 478.7 | 89.4 | 332 | 418 | 462 | 522 | 839 |
| CO_2_ (ppm) | Larnaka | outdoors | 359 | 410.3 | 44.2 | 347 | 392 | 404 | 420 | 549 |
| CO_2_ (ppm) | Limassol | indoors | 640 | 550.3 | 185.1 | 331 | 456 | 488 | 600 | 1828 |
| CO_2_ (ppm) | Limassol | outdoors | 346 | 446.4 | 75.4 | 354 | 404 | 420 | 461 | 711 |
| CO_2_ (ppm) | Nicosia | indoors | 823 | 519 | 107 | 303 | 451 | 494 | 573 | 1065 |
| CO_2_ (ppm) | Nicosia | outdoors | 488 | 431.8 | 50.2 | 358 | 407 | 416.5 | 442 | 708 |
| CO_2_ (ppm) | Paphos | indoors | 334 | 529.4 | 92 | 398 | 453 | 514.5 | 590 | 938 |
| CO_2_ (ppm) | Paphos | outdoors | 216 | 494.2 | 95.4 | 388 | 412 | 455 | 548 | 747 |
| RH (%) | Famagusta | indoors | 109 | 45.2 | 9.4 | 31 | 36.5 | 47 | 53 | 60.5 |
| RH (%) | Famagusta | outdoors | 82 | 40.8 | 9.4 | 29 | 33 | 42.2 | 48.5 | 58 |
| RH (%) | Larnaka | indoors | 544 | 54.3 | 7.3 | 33 | 49.4 | 56 | 59 | 67 |
| RH (%) | Larnaka | outdoors | 359 | 46.4 | 9.3 | 19 | 39.5 | 46.5 | 53.8 | 67.5 |
| RH (%) | Limassol | indoors | 640 | 49 | 7.5 | 21 | 43 | 48.8 | 55.1 | 65 |
| RH (%) | Limassol | outdoors | 346 | 43.4 | 10.3 | 21 | 35.6 | 42 | 52 | 62.5 |
| RH (%) | Nicosia | indoors | 823 | 37.8 | 5.5 | 22 | 33.5 | 38 | 41.5 | 50.5 |
| RH (%) | Nicosia | outdoors | 488 | 34.2 | 7.1 | 17 | 28.5 | 36.2 | 39.5 | 47 |
| RH (%) | Paphos | indoors | 334 | 50.5 | 8.5 | 35 | 45.5 | 50 | 54.5 | 75 |
| RH (%) | Paphos | outdoors | 216 | 46.2 | 8.6 | 30 | 39.5 | 44 | 50.6 | 69.5 |
| PM_1_ (ug/m^3^) | Famagusta | indoors | 641 | 8.6 | 1.9 | 4.9 | 6.8 | 8.5 | 10 | 14.2 |
| PM_1_ (ug/m^3^) | Famagusta | outdoors | 316 | 9.1 | 2.3 | 5 | 6.8 | 9.8 | 11.1 | 13.9 |
| PM_1_ (ug/m^3^) | Larnaka | indoors | 3947 | 10.5 | 4 | 2.1 | 7.2 | 10.1 | 13.1 | 23.6 |
| PM_1_ (ug/m^3^) | Larnaka | outdoors | 2258 | 10.9 | 4.6 | 2.7 | 7 | 10 | 13.6 | 23.5 |
| PM_1_ (ug/m^3^) | Limassol | indoors | 5289 | 12.8 | 5.2 | 1.9 | 8.6 | 11.9 | 17.1 | 42.5 |
| PM_1_ (ug/m^3^) | Limassol | outdoors | 2728 | 11.2 | 5.2 | 1.6 | 6.7 | 11.1 | 16.2 | 27.7 |
| PM_1_ (ug/m^3^) | Nicosia | indoors | 5972 | 9 | 4.1 | 0.2 | 6 | 8.7 | 11 | 32.7 |
| PM_1_ (ug/m^3^) | Nicosia | outdoors | 2885 | 9.7 | 4.4 | 2.7 | 6.7 | 9 | 10.8 | 26.4 |
| PM_1_ (ug/m^3^) | Paphos | indoors | 1430 | 14.2 | 4.1 | 4.3 | 11.5 | 14.5 | 16.1 | 30.3 |
| PM_1_ (ug/m^3^) | Paphos | outdoors | 641 | 11.5 | 3.7 | 3.5 | 8.2 | 12.5 | 14.6 | 27.2 |
| PM_10_ (ug/m^3^) | Famagusta | indoors | 641 | 13.3 | 2.8 | 7.5 | 10.9 | 13.3 | 15.4 | 25 |
| PM_10_ (ug/m^3^) | Famagusta | outdoors | 316 | 14.6 | 2.9 | 9.2 | 12 | 14.7 | 16.9 | 20.6 |
| PM_10_ (ug/m^3^) | Larnaka | indoors | 3947 | 15.5 | 5.9 | 3.7 | 11 | 14.5 | 19.2 | 51.5 |
| PM_10_ (ug/m^3^) | Larnaka | outdoors | 2258 | 17 | 7.4 | 4.6 | 11.1 | 15.5 | 21 | 42.4 |
| PM_10_ (ug/m^3^) | Limassol | indoors | 5289 | 18.2 | 7.2 | 2.9 | 12.8 | 16.9 | 23.8 | 42.2 |
| PM_10_ (ug/m^3^) | Limassol | outdoors | 2728 | 15.8 | 6.8 | 4 | 9.8 | 15.3 | 21.6 | 38.7 |
| PM_10_ (ug/m^3^) | Nicosia | indoors | 5972 | 13.9 | 6.6 | 1.4 | 9.2 | 13.3 | 16.4 | 50 |
| PM_10_ (ug/m^3^) | Nicosia | outdoors | 2885 | 15 | 8 | 4.3 | 10.1 | 13 | 16.6 | 47.4 |
| PM_10_ (ug/m^3^) | Paphos | indoors | 1430 | 22.2 | 5.3 | 7.8 | 19.6 | 21.5 | 24.4 | 52.3 |
| PM_10_ (ug/m^3^) | Paphos | outdoors | 641 | 18.6 | 5.2 | 7.2 | 14.4 | 19.3 | 22.4 | 46.8 |
| PM_2.5_ (ug/m^3^) | Famagusta | indoors | 641 | 12 | 2.5 | 6.9 | 9.6 | 12.1 | 13.8 | 21.2 |
| PM_2.5_ (ug/m^3^) | Famagusta | outdoors | 316 | 12.9 | 3.2 | 7.4 | 9.8 | 13.6 | 15.6 | 19.3 |
| PM_2.5_ (ug/m^3^) | Larnaka | indoors | 3947 | 14.1 | 5.5 | 2.9 | 9.7 | 13.3 | 17.6 | 39.6 |
| PM_2.5_ (ug/m^3^) | Larnaka | outdoors | 2258 | 15 | 6.8 | 3.3 | 9.5 | 13.4 | 18.6 | 41.6 |
| PM_2.5_ (ug/m^3^) | Limassol | indoors | 5289 | 16.8 | 7.4 | 2.2 | 11.6 | 15.3 | 21.9 | 98.8 |
| PM_2.5_ (ug/m^3^) | Limassol | outdoors | 2728 | 14.6 | 6.7 | 2.9 | 8.9 | 14 | 20.5 | 37.5 |
| PM_2.5_ (ug/m^3^) | Nicosia | indoors | 5972 | 12.4 | 6.1 | 0.7 | 8 | 12 | 15.1 | 44.9 |
| PM_2.5_ (ug/m^3^) | Nicosia | outdoors | 2885 | 13.1 | 6.2 | 3.8 | 9 | 12.1 | 14.7 | 37.4 |
| PM_2.5_ (ug/m^3^) | Paphos | indoors | 1430 | 19.9 | 4.8 | 6.5 | 17.9 | 19.8 | 21.5 | 39.8 |
| PM_2.5_ (ug/m^3^) | Paphos | outdoors | 641 | 16.1 | 4.8 | 5.7 | 12 | 17.2 | 19.6 | 36.5 |
| Temp. (°C) | Famagusta | indoors | 109 | 31.1 | 5.5 | 25 | 27 | 28.8 | 36.4 | 42.9 |
| Temp. (°C) | Famagusta | outdoors | 82 | 33.5 | 5.5 | 26 | 28.5 | 30.9 | 38.3 | 43.1 |
| Temp. (°C) | Larnaka | indoors | 544 | 29 | 3.2 | 26 | 26.7 | 27.5 | 31.7 | 39.9 |
| Temp. (°C) | Larnaka | outdoors | 359 | 30.7 | 3.3 | 26 | 28.7 | 29.7 | 31.6 | 44.5 |
| Temp. (°C) | Limassol | indoors | 640 | 29.5 | 2.4 | 26 | 28 | 29 | 31 | 41.8 |
| Temp. (°C) | Limassol | outdoors | 346 | 32 | 4.1 | 26 | 28.1 | 30.9 | 35.2 | 40.8 |
| Temp. (°C) | Nicosia | indoors | 823 | 29.2 | 1.2 | 26 | 28.3 | 29.2 | 30 | 33.1 |
| Temp. (°C) | Nicosia | outdoors | 488 | 30.6 | 2.1 | 26 | 29.7 | 30.7 | 31.6 | 36.2 |
| Temp. (°C) | Paphos | indoors | 334 | 29 | 2.2 | 25 | 27.2 | 28.8 | 30.5 | 33.7 |
| Temp. (°C) | Paphos | outdoors | 216 | 30.5 | 2.8 | 25 | 29.4 | 30.2 | 31.8 | 37.2 |
| VOCs (ppb) | Famagusta | indoors | 109 | 2664.4 | 9059.4 | 499 | 500 | 742 | 1990 | 65535 |
| VOCs (ppb) | Famagusta | outdoors | 82 | 3918.4 | 4741.6 | 499 | 506 | 1767 | 7837 | 21362 |
| VOCs (ppb) | Larnaka | indoors | 544 | 2232.4 | 5387.2 | 499 | 572 | 846.5 | 1440 | 64486 |
| VOCs (ppb) | Larnaka | outdoors | 359 | 6125.5 | 16610 | 499 | 555 | 642 | 974.5 | 65535 |
| VOCs (ppb) | Limassol | indoors | 640 | 2856.9 | 5132.8 | 499 | 732 | 1133 | 2118 | 55469 |
| VOCs (ppb) | Limassol | outdoors | 346 | 5836.5 | 11836 | 499 | 551 | 911.5 | 2343 | 57102 |
| VOCs (ppb) | Nicosia | indoors | 823 | 4963.8 | 12567 | 499 | 778 | 1312 | 2487 | 65535 |
| VOCs (ppb) | Nicosia | outdoors | 488 | 12847 | 22169 | 499 | 829 | 2088.5 | 6754 | 65535 |
| VOCs (ppb) | Paphos | indoors | 334 | 2241.4 | 3215 | 499 | 619 | 1234.5 | 2290 | 33283 |
| VOCs (ppb) | Paphos | outdoors | 216 | 14372 | 20136 | 499 | 595 | 2849 | 20152 | 65535 |

Table S6 Summary table for environmental parameters at schools during the whole sampling period (24-h or 48-h) (by district and location)

| **Parameter** | **District** | **Location** | **n** | **Mean** | **SD** | **Min** | **p25** | **Median** | **p75** | **Max** |
| --- | --- | --- | --- | --- | --- | --- | --- | --- | --- | --- |
| CO_2_ (ppm) | Famagusta | Indoors | 400 | 435.4 | 64.4 | 389 | 403 | 416 | 430.2 | 967 |
| CO_2_ (ppm) | Famagusta | Outdoors | 228 | 426.8 | 40.3 | 390 | 408 | 412 | 415 | 557 |
| CO_2_ (ppm) | Larnaka | Indoors | 2392 | 440.1 | 79.3 | 254 | 395 | 432 | 479 | 839 |
| CO_2_ (ppm) | Larnaka | Outdoors | 1342 | 401.1 | 34.6 | 345 | 364 | 406 | 417 | 549 |
| CO_2_ (ppm) | Limassol | Indoors | 2984 | 483.7 | 130.4 | 261 | 418 | 442 | 533 | 1828 |
| CO_2_ (ppm) | Limassol | Outdoors | 1538 | 426.5 | 54.9 | 346 | 405 | 420 | 433 | 774 |
| CO_2_ (ppm) | Nicosia | Indoors | 3654 | 464.3 | 89.4 | 300 | 408 | 447 | 507 | 1065 |
| CO_2_ (ppm) | Nicosia | Outdoors | 1980 | 421.7 | 32.8 | 356 | 409 | 419 | 430.2 | 708 |
| CO_2_ (ppm) | Paphos | Indoors | 1092 | 468.7 | 84.4 | 342 | 412 | 448 | 500 | 1055 |
| CO_2_ (ppm) | Paphos | Outdoors | 578 | 445.8 | 69.8 | 388 | 410 | 421 | 435 | 747 |
| PM_1_ (ug/m^3^) | Famagusta | Indoors | 2837 | 8.2 | 2.3 | 3.6 | 6.7 | 8.4 | 9.8 | 18.3 |
| PM_1_ (ug/m^3^) | Famagusta | Outdoors | 1393 | 8.9 | 2.7 | 3.7 | 6.4 | 9.4 | 10.9 | 21.5 |
| PM_1_ (ug/m^3^) | Larnaka | Indoors | 13459 | 10.1 | 4 | 1.5 | 7.4 | 10.2 | 12.9 | 23.6 |
| PM_1_ (ug/m^3^) | Larnaka | Outdoors | 9856 | 10.2 | 4.5 | 1.4 | 6.5 | 9.7 | 13.3 | 26.7 |
| PM_1_ (ug/m^3^) | Limassol | Indoors | 16860 | 12.7 | 5.7 | 1.9 | 8 | 12.2 | 16.3 | 42.5 |
| PM_1_ (ug/m^3^) | Limassol | Outdoors | 11451 | 11.5 | 5.2 | 1.4 | 7 | 10.8 | 15.8 | 35.9 |
| PM_1_ (ug/m^3^) | Nicosia | Indoors | 25819 | 8.5 | 3.4 | 0.2 | 5.9 | 8.2 | 10.4 | 32.2 |
| PM_1_ (ug/m^3^) | Nicosia | Outdoors | 12816 | 9.9 | 4.6 | 1.2 | 6.8 | 9.2 | 12.1 | 58.2 |
| PM_1_ (ug/m^3^) | Paphos | Indoors | 6654 | 14.6 | 4.3 | 4.3 | 11 | 14.7 | 17.4 | 30.3 |
| PM_1_ (ug/m^3^) | Paphos | Outdoors | 2813 | 12.9 | 4.7 | 3.1 | 8.9 | 12.7 | 16.2 | 37.4 |
| PM_10_ (ug/m^3^) | Famagusta | Indoors | 2837 | 12.2 | 3.3 | 5.6 | 9.8 | 12.4 | 14.5 | 25 |
| PM_10_ (ug/m^3^) | Famagusta | Outdoors | 1393 | 14.4 | 3.8 | 6.2 | 11.5 | 14.7 | 16.9 | 46.8 |
| PM_10_ (ug/m^3^) | Larnaka | Indoors | 13459 | 14.6 | 6.4 | 2.9 | 10.7 | 14 | 18.5 | 51.5 |
| PM_10_ (ug/m^3^) | Larnaka | Outdoors | 9856 | 15.4 | 7.3 | 2.4 | 9.6 | 14.3 | 19.8 | 49.3 |
| PM_10_ (ug/m^3^) | Limassol | Indoors | 16860 | 17.9 | 8.7 | 2.9 | 11.4 | 16.9 | 22.3 | 63.2 |
| PM_10_ (ug/m^3^) | Limassol | Outdoors | 11451 | 16 | 7.2 | 3.4 | 10.1 | 14.9 | 21.2 | 71.1 |
| PM_10_ (ug/m^3^) | Nicosia | Indoors | 25819 | 12.5 | 6 | 1.4 | 8.6 | 11.8 | 15.1 | 136.9 |
| PM_10_ (ug/m^3^) | Nicosia | Outdoors | 12816 | 14.9 | 7.8 | 2.1 | 9.7 | 13.3 | 17.8 | 98.2 |
| PM_10_ (ug/m^3^) | Paphos | Indoors | 6654 | 21.1 | 9.2 | 7.8 | 15.2 | 20.2 | 23.6 | 173.8 |
| PM_10_ (ug/m^3^) | Paphos | Outdoors | 2813 | 18.4 | 7 | 6.9 | 12.5 | 17.5 | 22.5 | 67.2 |
| PM_2.5_ (ug/m^3^) | Famagusta | Indoors | 2837 | 11.2 | 3.1 | 5.2 | 9 | 11.6 | 13.2 | 26 |
| PM_2.5_ (ug/m^3^) | Famagusta | Outdoors | 1393 | 12.7 | 3.8 | 5.4 | 9.4 | 13.2 | 15.6 | 34.6 |
| PM_2.5_ (ug/m^3^) | Larnaka | Indoors | 13459 | 13.6 | 5.6 | 2.6 | 9.9 | 13.4 | 17.4 | 45.1 |
| PM_2.5_ (ug/m^3^) | Larnaka | Outdoors | 9856 | 13.7 | 6.6 | 2.1 | 8.4 | 12.7 | 18 | 41.6 |
| PM_2.5_ (ug/m^3^) | Limassol | Indoors | 16860 | 16.8 | 8.5 | 2.2 | 10.5 | 15.9 | 21 | 98.8 |
| PM_2.5_ (ug/m^3^) | Limassol | Outdoors | 11451 | 14.9 | 7 | 2.4 | 9.1 | 13.8 | 20 | 55.7 |
| PM_2.5_ (ug/m^3^) | Nicosia | Indoors | 25819 | 11.5 | 5.4 | 0.7 | 7.8 | 10.9 | 14.1 | 95.8 |
| PM_2.5_ (ug/m^3^) | Nicosia | Outdoors | 12816 | 13.3 | 6.4 | 1.9 | 8.8 | 12.3 | 16.1 | 85.9 |
| PM_2.5_ (ug/m^3^) | Paphos | Indoors | 6654 | 19.6 | 7.7 | 6.5 | 14.2 | 18.9 | 22.1 | 83.7 |
| PM_2.5_ (ug/m^3^) | Paphos | Outdoors | 2813 | 16.9 | 6.2 | 5.7 | 11.5 | 16.5 | 20.4 | 55.5 |
| RH (%) | Famagusta | Indoors | 400 | 49.3 | 6.1 | 30.5 | 47 | 51 | 53 | 60.5 |
| RH (%) | Famagusta | Outdoors | 228 | 51.8 | 12.1 | 28.5 | 47.2 | 54.5 | 59 | 76 |
| RH (%) | Larnaka | Indoors | 2392 | 53.5 | 5.8 | 33 | 49 | 53.5 | 58 | 68.5 |
| RH (%) | Larnaka | Outdoors | 1342 | 52.3 | 12.4 | 18.5 | 42.5 | 54 | 62 | 79 |
| RH (%) | Limassol | Indoors | 2984 | 49.1 | 6.7 | 21 | 44.5 | 50 | 54.5 | 65 |
| RH (%) | Limassol | Outdoors | 1538 | 47.9 | 12.4 | 20.5 | 38.5 | 46 | 57.5 | 78 |
| RH (%) | Nicosia | Indoors | 3654 | 41 | 5.9 | 18 | 37.5 | 41.5 | 45 | 70.5 |
| RH (%) | Nicosia | Outdoors | 1980 | 48.6 | 16.7 | 16.5 | 35.5 | 45.5 | 63.5 | 81 |
| RH (%) | Paphos | Indoors | 1092 | 53.7 | 6.8 | 34.5 | 50.5 | 54 | 57.5 | 75 |
| RH (%) | Paphos | Outdoors | 578 | 52.8 | 11.3 | 30 | 44 | 52 | 61 | 79 |
| Temp. (°C) | Famagusta | Indoors | 400 | 27.8 | 3.7 | 24.4 | 25.7 | 27.0 | 28.1 | 42.9 |
| Temp. (°C) | Famagusta | Outdoors | 228 | 27.0 | 6.4 | 17.1 | 22.8 | 26.2 | 29.1 | 43.1 |
| Temp. (°C) | Larnaka | Indoors | 2392 | 28.5 | 2.8 | 24.9 | 26.3 | 27.4 | 30.1 | 39.9 |
| Temp. (°C) | Larnaka | Outdoors | 1342 | 28.0 | 4.2 | 19.3 | 24.6 | 28.1 | 30.8 | 44.5 |
| Temp. (°C) | Limassol | Indoors | 2984 | 29.2 | 2.7 | 25.1 | 27 | 28.8 | 31 | 41.9 |
| Temp. (°C) | Limassol | Outdoors | 1538 | 28.8 | 4.6 | 20.6 | 25.3 | 27.7 | 32 | 40.8 |
| Temp. (°C) | Nicosia | Indoors | 3654 | 28.7 | 1.6 | 23.3 | 27.8 | 28.6 | 29.4 | 35 |
| Temp. (°C) | Nicosia | Outdoors | 1980 | 26.1 | 4.4 | 17 | 22.3 | 25.6 | 30 | 41.2 |
| Temp. (°C) | Paphos | Indoors | 1092 | 27.8 | 2.1 | 25 | 26.4 | 27.0 | 29.8 | 33.7 |
| Temp. (°C) | Paphos | Outdoors | 578 | 27.7 | 4 | 21.3 | 24.5 | 27.2 | 30.4 | 39.4 |
| VOCs (ppb) | Famagusta | Indoors | 400 | 1496.2 | 5267 | 499 | 521 | 599 | 850.2 | 65535 |
| VOCs (ppb) | Famagusta | Outdoors | 228 | 2643.2 | 3623 | 499 | 572.2 | 1276.5 | 2639 | 21362 |
| VOCs (ppb) | Larnaka | Indoors | 2392 | 2375.8 | 4915 | 499 | 633 | 996.5 | 1658.2 | 65535 |
| VOCs (ppb) | Larnaka | Outdoors | 1342 | 4139.5 | 9343 | 499 | 650.5 | 1406.5 | 3199.5 | 65535 |
| VOCs (ppb) | Limassol | Indoors | 2984 | 1430.3 | 2756 | 499 | 571 | 768.5 | 1188.2 | 55469 |
| VOCs (ppb) | Limassol | Outdoors | 1538 | 2344.4 | 6014 | 499 | 610 | 961.5 | 1706.8 | 57102 |
| VOCs (ppb) | Nicosia | Indoors | 3654 | 1957.3 | 6468 | 342 | 557.2 | 712.5 | 1172.8 | 65535 |
| VOCs (ppb) | Nicosia | Outdoors | 1980 | 5076.2 | 12472 | 499 | 716.8 | 1400.5 | 3037.8 | 65535 |
| VOCs (ppb) | Paphos | Indoors | 1092 | 1398 | 2200 | 499 | 595 | 887 | 1301 | 33283 |
| VOCs (ppb) | Paphos | Outdoors | 578 | 6107.4 | 13883 | 499 | 536.2 | 825.5 | 2555.2 | 65535 |

Table S7 Summary table of environmental parameters in schools during school hours - school period mean values

| **Parameter** | **Class type** | **n** | **Mean** | **SD** | **Min** | **p25** | **Median** | **p75** | **Max** |
| --- | --- | --- | --- | --- | --- | --- | --- | --- | --- |
| PM_1_ (ug/m^3^) | typical | 1339 | 10.8 | 5.0 | 0.5 | 7.0 | 10.2 | 13.7 | 29.7 |
| PM_1_ (ug/m^3^) | atypical | 198 | 11.1 | 4.6 | 2.7 | 7.6 | 10.5 | 14.5 | 24.7 |
| PM_10_ (ug/m^3^) | typical | 1339 | 16.4 | 6.9 | 2.7 | 11.0 | 15.4 | 20.5 | 44.4 |
| PM_10_ (ug/m^3^) | atypical | 198 | 17.3 | 6.9 | 4.8 | 12.4 | 15.9 | 21.5 | 37.5 |
| PM_2.5_ (ug/m^3^) | typical | 1339 | 14.8 | 6.6 | 1.6 | 9.7 | 14.1 | 18.6 | 56.8 |
| PM_2.5_ (ug/m^3^) | atypical | 198 | 15.7 | 6.9 | 4.0 | 10.6 | 14.3 | 19.7 | 36.7 |
| VOCs (ppb) | typical | 1105 | 2820.3 | 7203.7 | 499.0 | 669.5 | 1037.8 | 1889.0 | 65535 |
| VOCs (ppb) | atypical | 299 | 2869.9 | 5585.5 | 499.0 | 690.9 | 1091.5 | 1999.4 | 55469 |
| CO_2_ (ppm) | typical | 1105 | 510.2 | 103.8 | 303.5 | 434.5 | 489.4 | 562.7 | 1052 |
| CO_2_ (ppm) | atypical | 299 | 535.8 | 225.5 | 332.0 | 432.0 | 466.0 | 553.8 | 1789 |
| RH (%) | typical | 1105 | 44.9 | 9.9 | 22.5 | 37.5 | 44.3 | 52.5 | 75.0 |
| RH (%) | atypical | 299 | 53.6 | 6.8 | 33.0 | 50.2 | 55.0 | 58.1 | 66.8 |
| Temp. (°C) | typical | 1105 | 29.3 | 2.3 | 25.4 | 27.5 | 28.9 | 30.3 | 41.8 |
| Temp. (°C) | atypical | 299 | 27.7 | 1.4 | 25.6 | 26.2 | 27.7 | 28.9 | 31.2 |
| VOCs (ppb) | typical | 1105 | 2820.3 | 7203.7 | 499.0 | 669.5 | 1037.8 | 1889.0 | 65535 |
| VOCs (ppb) | atypical | 299 | 2869.9 | 5585.5 | 499.0 | 690.9 | 1091.5 | 1999.4 | 55469 |

Table S8 Linear mixed effect models of particulate matter parameters measured in atypical school classrooms (indoors) during school hours regressed on the outdoor levels and adjusted for indoor temperature and humidity levels, period type (break vs class time), school period (1-10), percentage of open windows, open doors and fans in use, recent paint inside the classroom (yes vs no) and chlorine use frequency during classrooms’ cleaning (five times per week vs less or equal to three times per week) .

|  | **Indoor PM_1_** | **Indoor PM_2.5_** | **Indoor PM_10_** |
| --- | --- | --- | --- |
| *Predictors* | *Estimate (95% CI)* | *Estimate (95% CI)* | *Estimate (95% CI)* |
| Outdoor PM_1_ | 0.958 ^***^ (0.943 – 0.973) |  |  |
| Indoor temperature | 0.422 ^***^ (0.247 – 0.597) | 0.566 ^***^ (0.365 – 0.767) | 0.580 ^***^ (0.345 – 0.815) |
| Indoor humidity | -0.118 ^***^ (-0.132 – -0.104) | -0.170 ^***^ (-0.188 – -0.152) | -0.206 ^***^ (-0.227 – -0.184) |
| School break | -0.484 ^***^ (-0.568 – -0.399) | -0.526 ^***^ (-0.634 – -0.418) | -0.232 ^***^ (-0.359 – -0.106) |
| School period | 0.263 ^***^ (0.247 – 0.278) | 0.365 ^***^ (0.345 – 0.385) | 0.414 ^***^ (0.391 – 0.437) |
| % open windows | -0.019 ^***^ (-0.023 – -0.015) | -0.032 ^***^ (-0.037 – -0.027) | -0.041 ^***^ (-0.047 – -0.035) |
| % fans in use | -0.000  (-0.003 – 0.002) | 0.005 ^***^ (0.002 – 0.007) | 0.004 ^**^ (0.001 – 0.007) |
| % open doors | 0.003  (-0.001 – 0.008) | 0.006  (-0.000 – 0.012) | 0.006  (-0.001 – 0.012) |
| Recent painting of classroom | -0.193  (-2.773 – 2.386) | -0.738  (-4.231 – 2.755) | -0.788  (-4.278 – 2.703) |
| Five times a week use of chlorine during classroom cleaning | -0.853  (-3.446 – 1.740) | -1.288  (-4.794 – 2.217) | -2.356  (-5.863 – 1.150) |
| Outdoor PM_2.5_ |  | 0.918 ^***^ (0.903 – 0.933) |  |
| Outdoor PM_10_ |  |  | 0.896 ^***^ (0.879 – 0.913) |
| Residual variance | 0.61 | 1.05 | 1.41 |
| Classroom-level random intercept variance | 5.04 | 9.27 | 9.23 |
| ICC | 0.89 | 0.89 | 0.86 |
| Number of schools | 9 | 9 | 9 |
| Number of classrooms | 2 | 2 | 2 |
| Number of measurements | 3005 | 3117 | 3051 |

Models’ details:

(a) Environmental parameters (indoors and outdoors) are school-period averages.

(b) Random intercepts for the repeated measurements within classrooms, and classrooms nested within schools, with unstructured covariance matrix.

(c) **p<0.05   ** p<0.01   *** p<0.001*

Table S9 Linear mixed effect models results of indoor air quality for MCF parameters measured in atypical school classrooms (indoors) during school hours regressed on the outdoor levels and adjusted for period type (break vs class time), school period (1-10), percentage of open windows, open doors and fans in use, recent paint inside the classroom (yes vs no) and chlorine use frequency during classrooms’ cleaning (five times per week vs less or equal to three times per week).

|  | **Indoor temperature** | **Indoor humidity** | **Indoor CO_2_** | **Indoor VOCs** |
| --- | --- | --- | --- | --- |
| *Predictors* | *Estimate (95% CI)* | *Estimate (95% CI)* | *Estimate (95% CI)* | *Estimate (95% CI)* |
| Outdoor temperature | -0.050 ^***^ (-0.072 – -0.027) |  |  |  |
| Indoor humidity | -0.032 ^***^ (-0.039 – -0.025) |  |  |  |
| School break | 0.005  (-0.047 – 0.058) | -0.231  (-0.771 – 0.310) | -0.003  (-0.058 – 0.053) | -0.147  (-0.465 – 0.170) |
| School period | 0.039 ^***^ (0.027 – 0.051) | 0.024  (-0.068 – 0.117) | -0.023 ^***^ (-0.032 – -0.014) | 0.023  (-0.023 – 0.069) |
| % open windows | 0.008 ^***^ (0.006 – 0.011) | 0.041 ^***^ (0.020 – 0.062) | 0.001  (-0.001 – 0.003) | 0.003  (-0.004 – 0.010) |
| % fans in use | 0.003 ^***^ (0.002 – 0.004) | 0.019 ^***^ (0.011 – 0.028) | -0.000  (-0.001 – 0.001) | 0.000  (-0.004 – 0.004) |
| % open doors | 0.001  (-0.002 – 0.003) | 0.026 ^*^ (0.002 – 0.050) | 0.003 ^*^ (0.000 – 0.005) | -0.004  (-0.012 – 0.003) |
| Recent painting of classroom | 1.061  (-0.312 – 2.434) | -0.586  (-4.015 – 2.843) | -0.053  (-0.343 – 0.236) | 0.195  (-0.340 – 0.730) |
| Five times a week use of chlorine during classroom cleaning | 1.753 ^*^ (0.378 – 3.128) | -1.701  (-5.297 – 1.894) | -0.052  (-0.342 – 0.239) | 0.196  (-0.343 – 0.735) |
| Outdoor humidity |  | 0.347 ^***^ (0.300 – 0.394) |  |  |
| Indoor temperature |  | -1.830 ^***^ (-2.555 – -1.105) |  |  |
| Outdoor CO_2_ |  |  | 1.425 ^***^ (1.123 – 1.728) |  |
| Outdoor VOCs |  |  |  | 0.251 ^**^ (0.065 – 0.436) |
| Residual variance | 0.04 | 3.88 | 0.02 | 0.68 |
| Classroom-level random intercept variance | 1.46 | 8.37 | 0.06 | 0.14 |
| ICC | 0.98 | 0.68 | 0.74 | 0.17 |
| Number of schools | 9 | 9 | 9 | 9 |
| Number of classrooms | 2 | 2 | 2 | 2 |
| Number of measurements | 507 | 507 | 271 | 271 |

Models’ details:

(a) Environmental parameters (indoors and outdoors) are school-period averages.

(b) CO_2_ and VOCs (indoors and outdoors) are log-transformed.

(c) Random intercepts for the repeated measurements within classrooms, and classrooms nested within schools, with unstructured covariance matrix.

(d) **p<0.05   ** p<0.01   *** p<0.001*

Table S10 Percent exceedances of select indoor air quality indicators during school hours per district and degree of urbanization, based on international cut-offs. For PM_2.5_ and PM_10_, 24–h data were used.

| **Parameter** | **District** | **Degree of urbanization** | **n** | **Categories** | **# values per category** | **% values per category** |
| --- | --- | --- | --- | --- | --- | --- |
| Temperature | Famagusta | 2 | 109 | >27°C | 82 | 75 |
| Temperature | Famagusta | 2 | 109 | 22°C-27°C | 27 | 25 |
| Temperature | Larnaka | 1 | 307 | >27°C | 166 | 54 |
| Temperature | Larnaka | 1 | 307 | 22°C-27°C | 141 | 46 |
| Temperature | Larnaka | 2 | 237 | >27°C | 206 | 87 |
| Temperature | Larnaka | 2 | 237 | 22°C-27°C | 31 | 13 |
| Temperature | Limassol | 1 | 446 | >27°C | 360 | 81 |
| Temperature | Limassol | 1 | 446 | 22°C-27°C | 86 | 19 |
| Temperature | Limassol | 2 | 194 | >27°C | 194 | 100 |
| Temperature | Limassol | 2 | 194 | 22°C-27°C | 0 | 0 |
| Temperature | Nicosia | 1 | 697 | >27°C | 675 | 97 |
| Temperature | Nicosia | 1 | 697 | 22°C-27°C | 22 | 3 |
| Temperature | Nicosia | 2 | 126 | >27°C | 126 | 100 |
| Temperature | Nicosia | 2 | 126 | 22°C-27°C | 0 | 0 |
| Temperature | Paphos | 2 | 334 | >27°C | 271 | 81 |
| Temperature | Paphos | 2 | 334 | 22°C-27°C | 63 | 19 |
| RH | Famagusta | 2 | 109 | <40% | 38 | 35 |
| RH | Famagusta | 2 | 109 | >60% | 1 | 1 |
| RH | Famagusta | 2 | 109 | 40% - 60% | 70 | 64 |
| RH | Larnaka | 1 | 307 | <40% | 1 | 0 |
| RH | Larnaka | 1 | 307 | >60% | 85 | 28 |
| RH | Larnaka | 1 | 307 | 40% - 60% | 221 | 72 |
| RH | Larnaka | 2 | 237 | <40% | 25 | 11 |
| RH | Larnaka | 2 | 237 | >60% | 33 | 14 |
| RH | Larnaka | 2 | 237 | 40% - 60% | 179 | 76 |
| RH | Limassol | 1 | 446 | <40% | 34 | 8 |
| RH | Limassol | 1 | 446 | >60% | 28 | 6 |
| RH | Limassol | 1 | 446 | 40% - 60% | 384 | 86 |
| RH | Limassol | 2 | 194 | <40% | 36 | 19 |
| RH | Limassol | 2 | 194 | >60% | 0 | 0 |
| RH | Limassol | 2 | 194 | 40% - 60% | 158 | 81 |
| RH | Nicosia | 1 | 697 | <40% | 415 | 60 |
| RH | Nicosia | 1 | 697 | >60% | 0 | 0 |
| RH | Nicosia | 1 | 697 | 40% - 60% | 282 | 40 |
| RH | Nicosia | 2 | 126 | <40% | 102 | 81 |
| RH | Nicosia | 2 | 126 | >60% | 0 | 0 |
| RH | Nicosia | 2 | 126 | 40% - 60% | 24 | 19 |
| RH | Paphos | 2 | 334 | <40% | 33 | 10 |
| RH | Paphos | 2 | 334 | >60% | 40 | 12 |
| RH | Paphos | 2 | 334 | 40% - 60% | 261 | 78 |
| CO_2_ | Famagusta | 2 | 109 | <= 800 ppm | 107 | 98 |
| CO_2_ | Famagusta | 2 | 109 | >1350 ppm | 0 | 0 |
| CO_2_ | Famagusta | 2 | 109 | 800-1350 ppm | 2 | 2 |
| CO_2_ | Larnaka | 1 | 307 | <= 800 ppm | 305 | 99 |
| CO_2_ | Larnaka | 1 | 307 | >1350 ppm | 0 | 0 |
| CO_2_ | Larnaka | 1 | 307 | 800-1350 ppm | 2 | 1 |
| CO_2_ | Larnaka | 2 | 237 | <= 800 ppm | 237 | 100 |
| CO_2_ | Larnaka | 2 | 237 | >1350 ppm | 0 | 0 |
| CO_2_ | Larnaka | 2 | 237 | 800-1350 ppm | 0 | 0 |
| CO_2_ | Limassol | 1 | 446 | <= 800 ppm | 417 | 93 |
| CO_2_ | Limassol | 1 | 446 | >1350 ppm | 11 | 2 |
| CO_2_ | Limassol | 1 | 446 | 800-1350 ppm | 18 | 4 |
| CO_2_ | Limassol | 2 | 194 | <= 800 ppm | 191 | 98 |
| CO_2_ | Limassol | 2 | 194 | >1350 ppm | 0 | 0 |
| CO_2_ | Limassol | 2 | 194 | 800-1350 ppm | 3 | 2 |
| CO_2_ | Nicosia | 1 | 697 | <= 800 ppm | 680 | 98 |
| CO_2_ | Nicosia | 1 | 697 | >1350 ppm | 0 | 0 |
| CO_2_ | Nicosia | 1 | 697 | 800-1350 ppm | 17 | 2 |
| CO_2_ | Nicosia | 2 | 126 | <= 800 ppm | 122 | 97 |
| CO_2_ | Nicosia | 2 | 126 | >1350 ppm | 0 | 0 |
| CO_2_ | Nicosia | 2 | 126 | 800-1350 ppm | 4 | 3 |
| CO_2_ | Paphos | 2 | 334 | <= 800 ppm | 331 | 99 |
| CO_2_ | Paphos | 2 | 334 | >1350 ppm | 0 | 0 |
| CO_2_ | Paphos | 2 | 334 | 800-1350 ppm | 3 | 1 |
| PM_2.5_ | Famagusta | 2 | 4 | <=15 ug/m3 | 4 | 100 |
| PM_2.5_ | Famagusta | 2 | 4 | >15 ug/m3 | 0 | 0 |
| PM_2.5_ | Larnaka | 1 | 10 | <=15 ug/m3 | 10 | 100 |
| PM_2.5_ | Larnaka | 1 | 10 | >15 ug/m3 | 0 | 0 |
| PM_2.5_ | Larnaka | 2 | 6 | <=15 ug/m3 | 2 | 33 |
| PM_2.5_ | Larnaka | 2 | 6 | >15 ug/m3 | 4 | 67 |
| PM_2.5_ | Limassol | 1 | 19 | <=15 ug/m3 | 8 | 42 |
| PM_2.5_ | Limassol | 1 | 19 | >15 ug/m3 | 11 | 58 |
| PM_2.5_ | Limassol | 2 | 4 | <=15 ug/m3 | 2 | 50 |
| PM_2.5_ | Limassol | 2 | 4 | >15 ug/m3 | 2 | 50 |
| PM_2.5_ | Nicosia | 1 | 25 | <=15 ug/m3 | 23 | 92 |
| PM_2.5_ | Nicosia | 1 | 25 | >15 ug/m3 | 2 | 8 |
| PM_2.5_ | Nicosia | 2 | 4 | <=15 ug/m3 | 4 | 100 |
| PM_2.5_ | Nicosia | 2 | 4 | >15 ug/m3 | 0 | 0 |
| PM_2.5_ | Paphos | 2 | 10 | <=15 ug/m3 | 2 | 20 |
| PM_2.5_ | Paphos | 2 | 10 | >15 ug/m3 | 8 | 80 |
| PM_10_ | Famagusta | 2 | 4 | <=45 ug/m3 | 4 | 100 |
| PM_10_ | Larnaka | 1 | 10 | <=45 ug/m3 | 10 | 100 |
| PM_10_ | Larnaka | 2 | 6 | <=45 ug/m3 | 6 | 100 |
| PM_10_ | Limassol | 1 | 19 | <=45 ug/m3 | 19 | 100 |
| PM_10_ | Limassol | 2 | 4 | <=45 ug/m3 | 4 | 100 |
| PM_10_ | Nicosia | 1 | 25 | <=45 ug/m3 | 25 | 100 |
| PM_10_ | Nicosia | 2 | 4 | <=45 ug/m3 | 4 | 100 |
| PM_10_ | Paphos | 2 | 10 | <=45 ug/m3 | 10 | 100 |

# **Figures**

Figure S1 Data analysis workflow

^
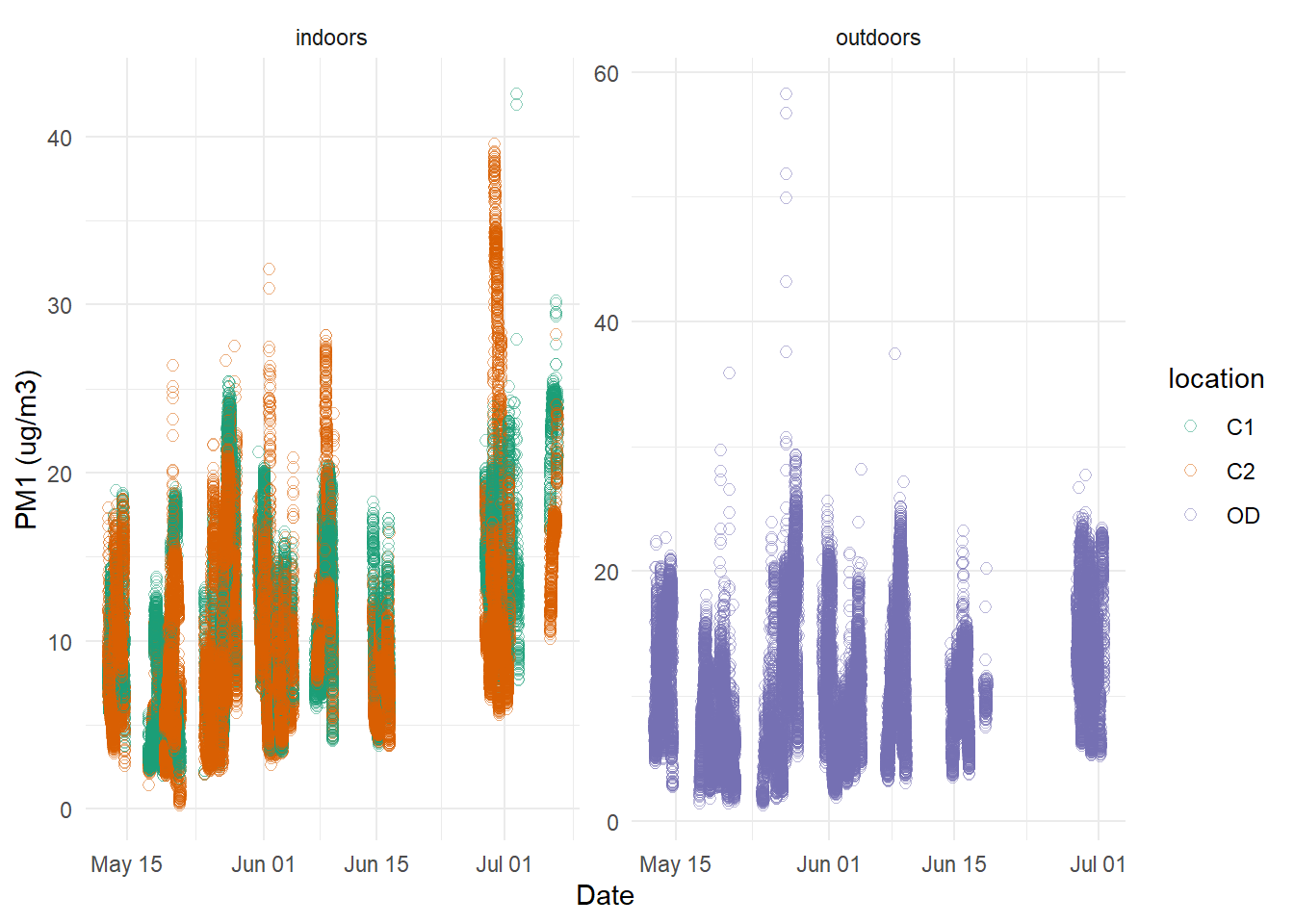

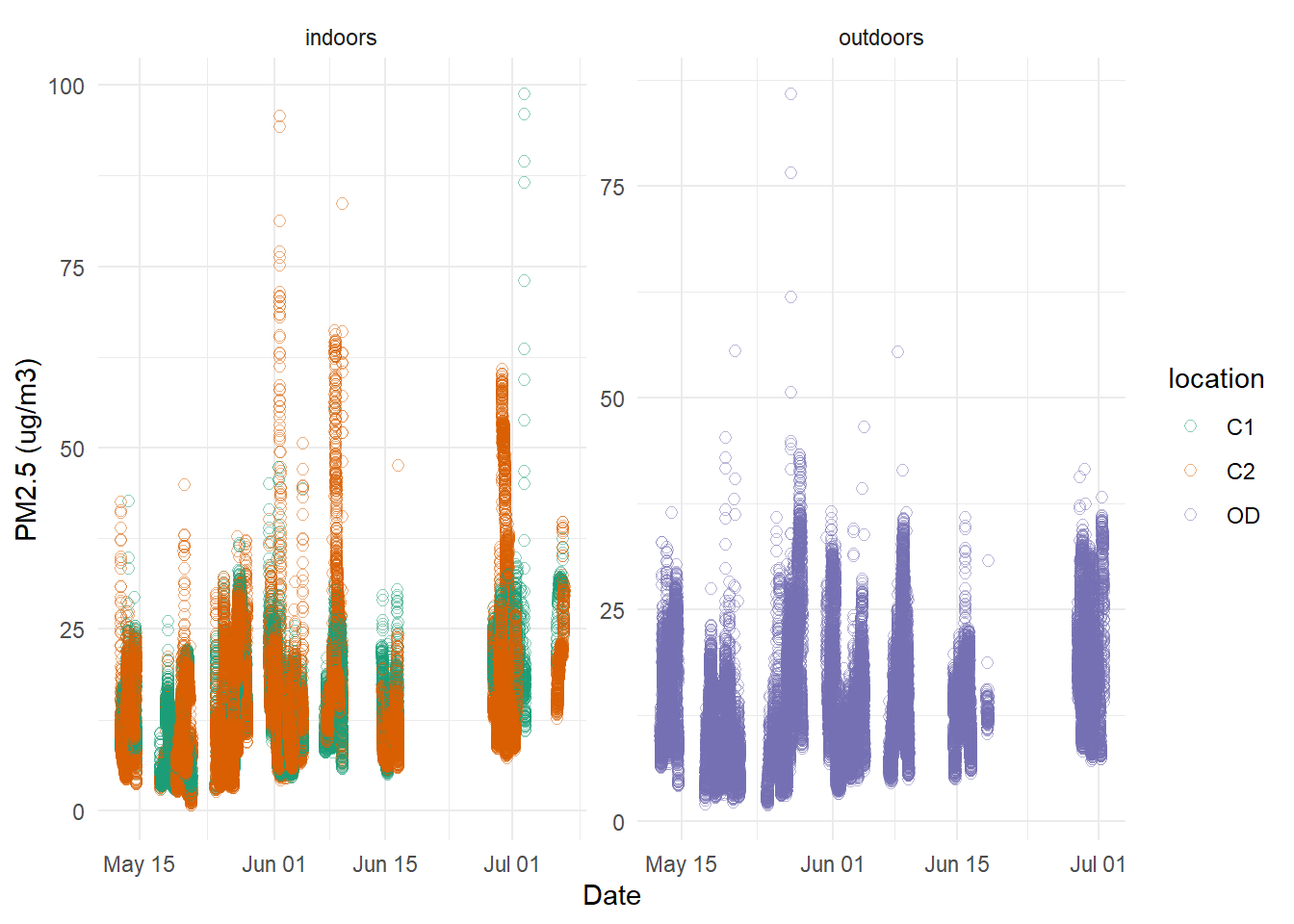

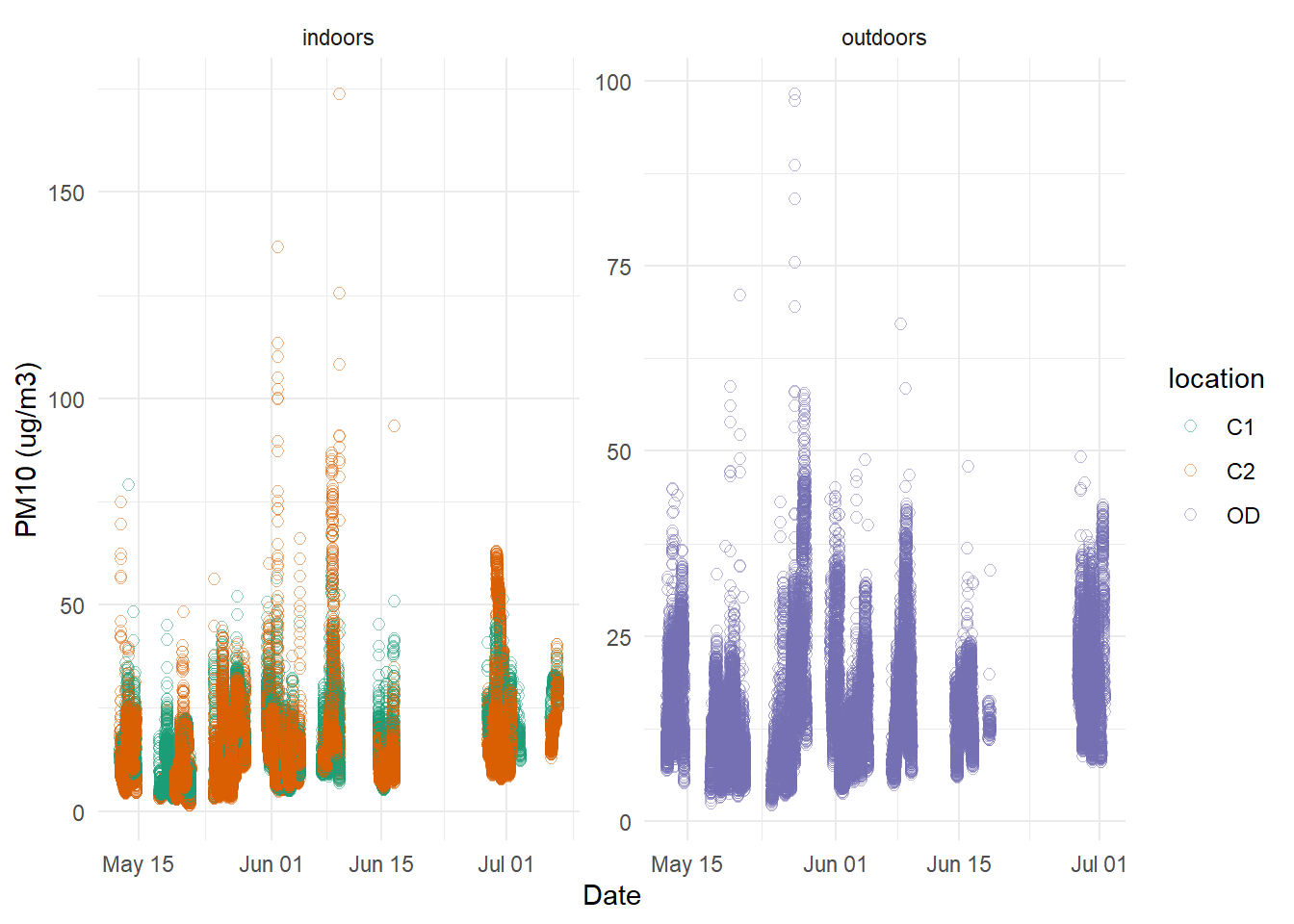

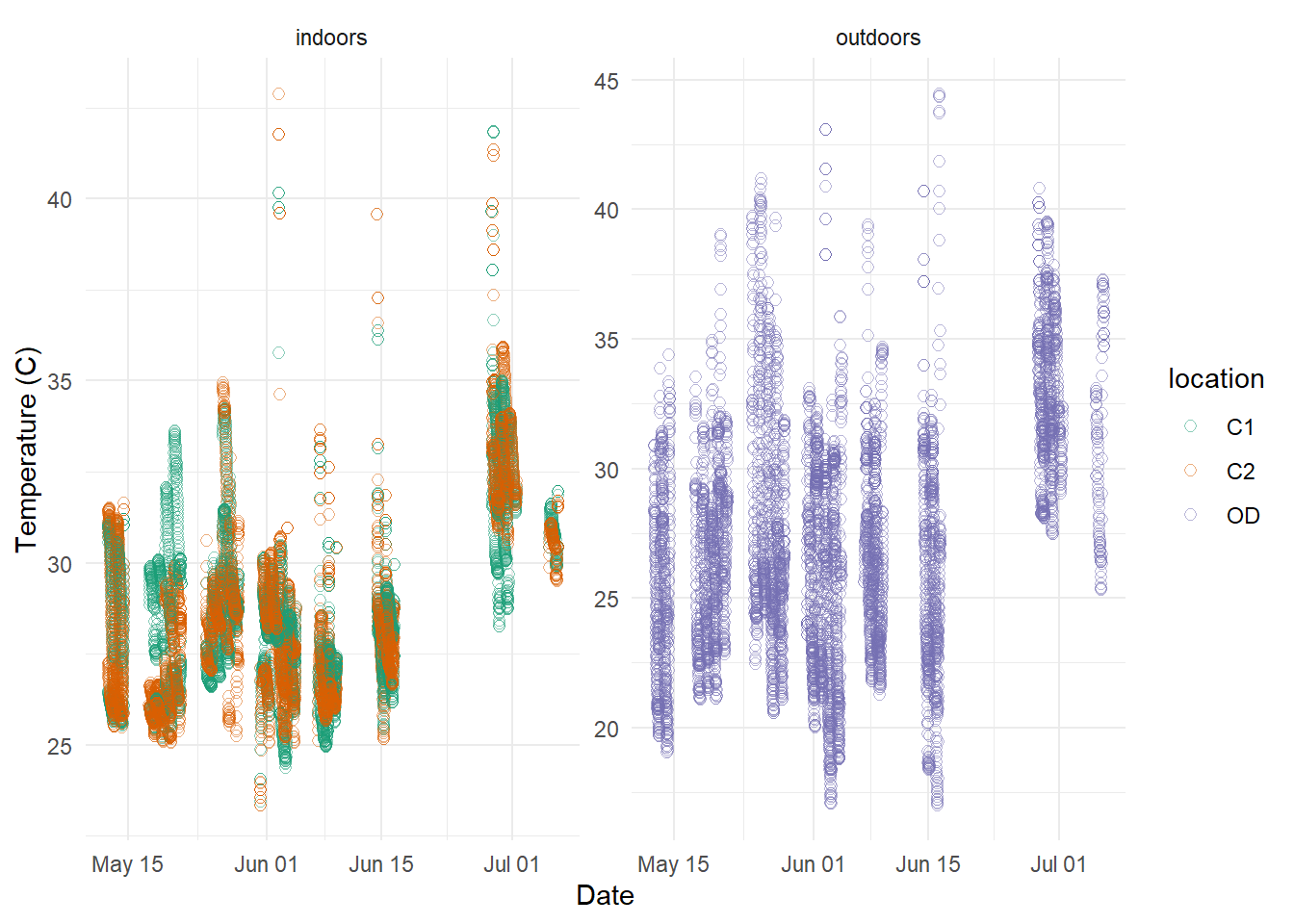

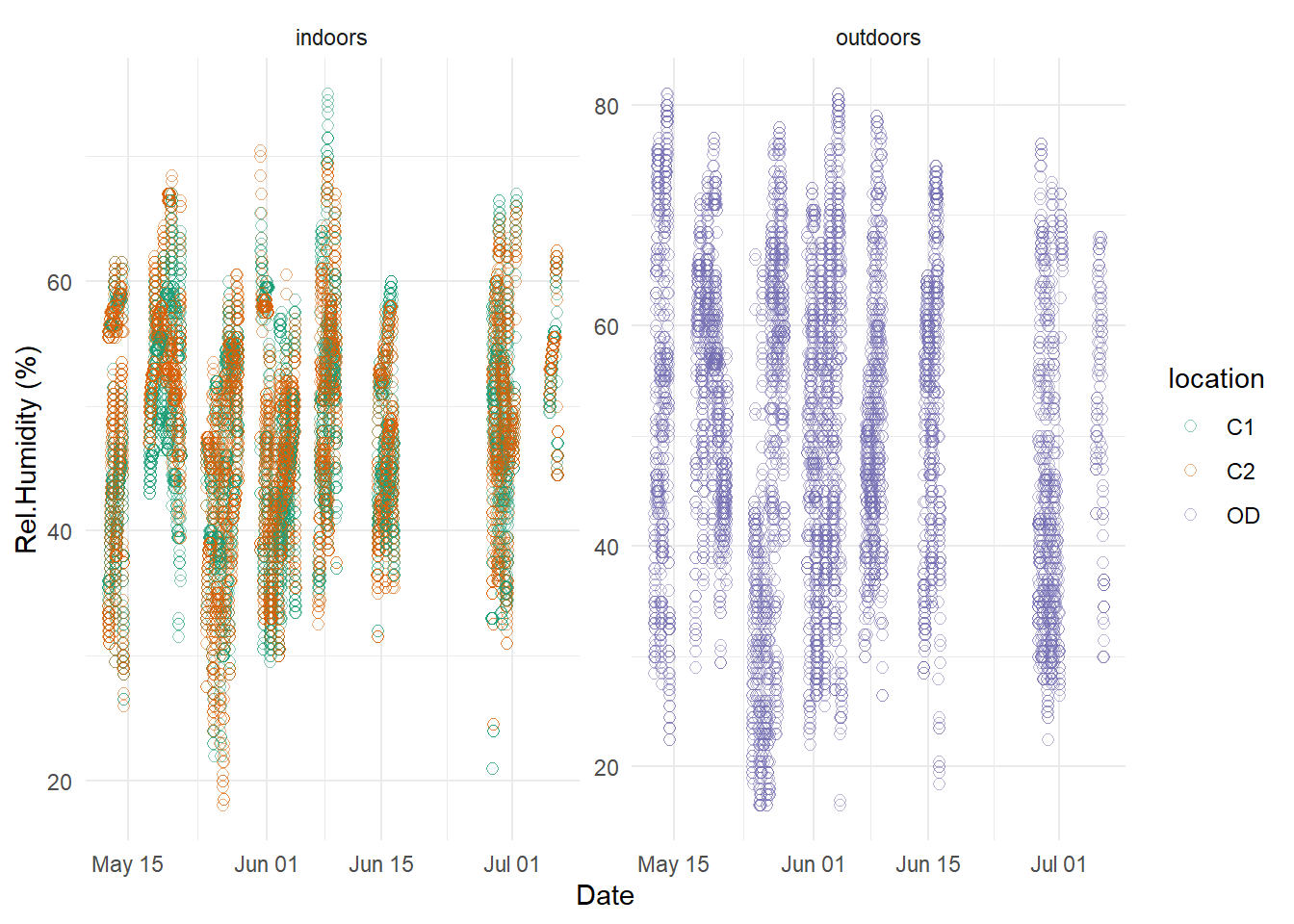

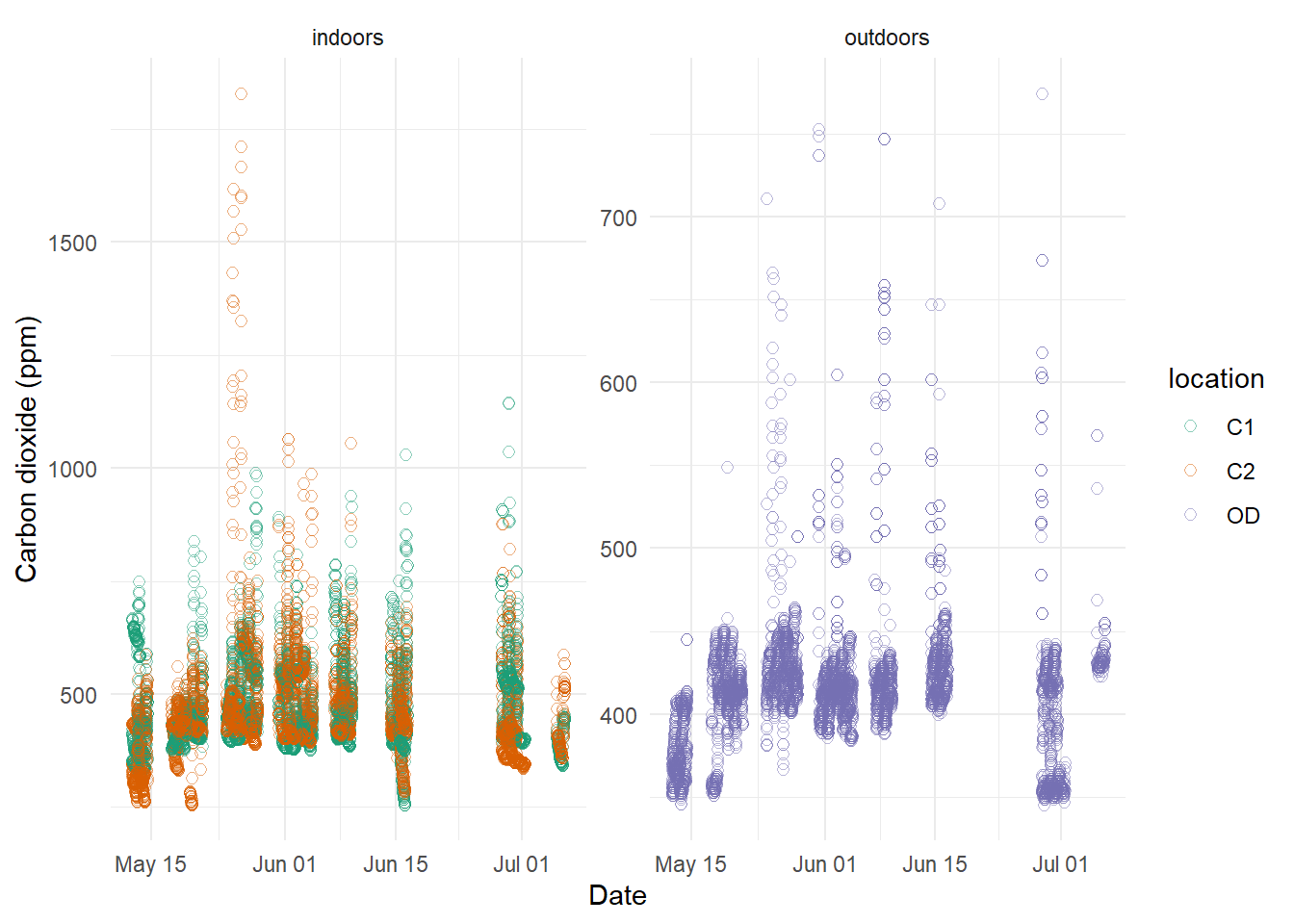

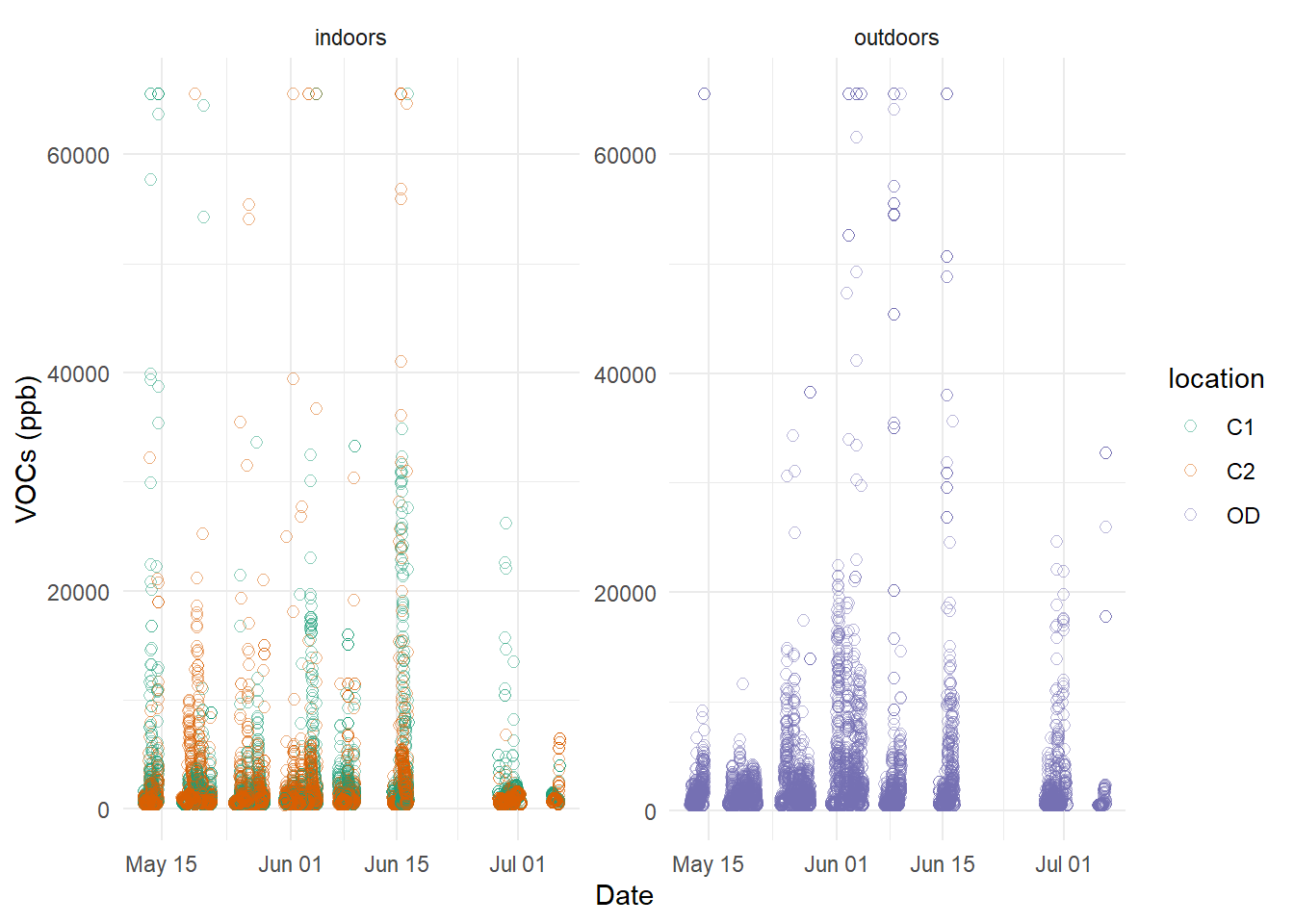
^

Figure S2 Time-series plots of environmental parameters (raw values) throughout the study period
